# Unexpected effect of catalyst concentration on photochemical CO_2_ reduction by *trans*(Cl)–Ru(bpy)(CO)_2_Cl_2_: new mechanistic insight into the CO/HCOO^–^ selectivity†
†Electronic supplementary information (ESI) available. See DOI: 10.1039/c5sc00199d



**DOI:** 10.1039/c5sc00199d

**Published:** 2015-03-12

**Authors:** Yusuke Kuramochi, Jun Itabashi, Kyohei Fukaya, Akito Enomoto, Makoto Yoshida, Hitoshi Ishida

**Affiliations:** a Department of Chemistry , Graduate School of Science , Kitasato University , 1-15-1 Kitasato, Minami-ku , Sagamihara , Kanagawa 252-0373 , Japan . Email: ishida@sci.kitasato-u.ac.jp; b Precursory Research for Embryonic Science (PRESTO) , Japan Science and Technology Agency (JST) , 4-1-8 Honcho , Kawaguchi , Saitama 332-0012 , Japan

## Abstract

Photochemical CO_2_ reduction catalysed by *trans*(Cl)–Ru(bpy)(CO)_2_Cl_2_ (bpy = 2,2′-bipyridine) efficiently produces carbon monoxide (CO) and formate (HCOO^–^) in *N*,*N*-dimethylacetamide (DMA)/water containing [Ru(bpy)_3_]^2+^ as a photosensitizer and 1-benzyl-1,4-dihydronicotinamide (BNAH) as an electron donor. We have unexpectedly found catalyst concentration dependence of the product ratio (CO/HCOO^–^) in the photochemical CO_2_ reduction: the ratio of CO/HCOO^–^ decreases with increasing catalyst concentration. The result has led us to propose a new mechanism in which HCOO^–^ is selectively produced by the formation of a Ru(i)–Ru(i) dimer as the catalyst intermediate. This reaction mechanism predicts that the Ru–Ru bond dissociates in the reaction of the dimer with CO_2_, and that the insufficient electron supply to the catalyst results in the dominant formation of HCOO^–^. The proposed mechanism is supported by the result that the time-course profiles of CO and HCOO^–^ in the photochemical CO_2_ reduction catalysed by [Ru(bpy)(CO)_2_Cl]_2_ (0.05 mM) are very similar to those of the reduction catalysed by *trans*(Cl)–Ru(bpy)(CO)_2_Cl_2_ (0.10 mM), and that HCOO^–^ formation becomes dominant under low-intensity light. The kinetic analyses based on the proposed mechanism could excellently reproduce the unusual catalyst concentration effect on the product ratio. The catalyst concentration effect observed in the photochemical CO_2_ reduction using [Ru(4dmbpy)_3_]^2+^ (4dmbpy = 4,4′-dimethyl-2,2′-bipyridine) instead of [Ru(bpy)_3_]^2+^ as the photosensitizer is also explained with the kinetic analyses, reflecting the smaller quenching rate constant of excited [Ru(4dmbpy)_3_]^2+^ by BNAH than that of excited [Ru(bpy)_3_]^2+^. We have further synthesized *trans*(Cl)–Ru(6Mes-bpy)(CO)_2_Cl_2_ (6Mes-bpy = 6,6′-dimesityl-2,2′-bipyridine), which bears bulky substituents at the 6,6′-positions in the 2,2′-bipyridyl ligand, so that the ruthenium complex cannot form the dimer due to the steric hindrance. We have found that this ruthenium complex selectively produces CO, which strongly supports the catalytic mechanism proposed in this work.

## Abstract 2

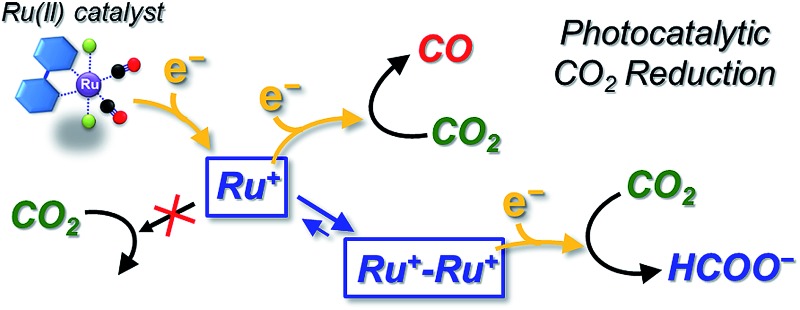
We found catalyst concentration dependence of the product ratio in the photochemical reduction of CO_2_, and proposed a new mechanism involving a Ru(i)–Ru(i) dimer intermediate.

## Introduction

**Scheme 1:**
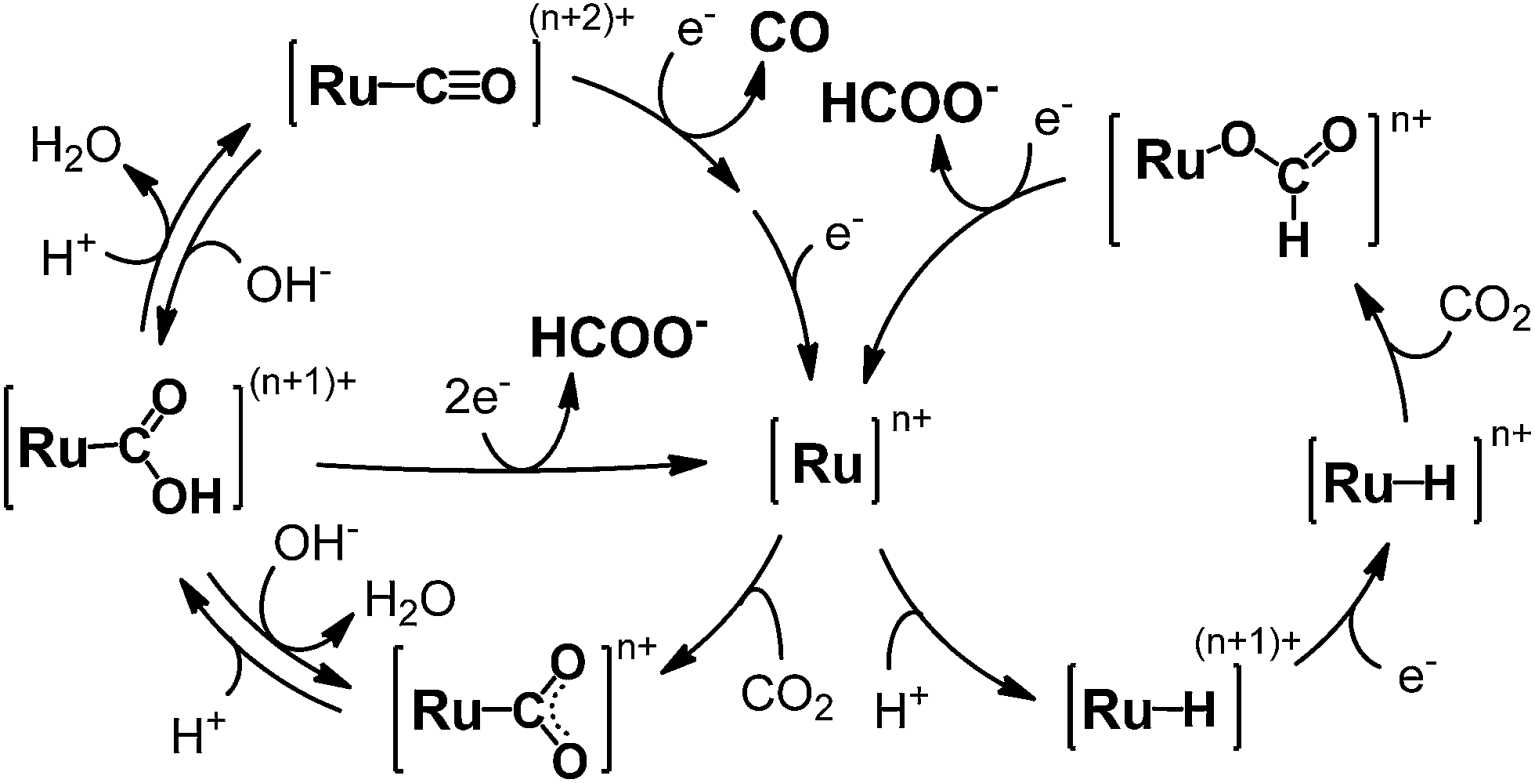
Combined mechanisms^48^ proposed by Tanaka *et al.* ^69^ and Meyer *et al.* ^50^

Photocatalytic CO_2_ reduction represents a major concern in relation to the construction of artificial photosynthetic systems and solar fuels, which are relevant to the solution of the fossil fuel shortage and the global warming problems.^1–4^ Until now, many metal complexes have been investigated for CO_2_ reduction catalysis.^5–15^ As metal complexes for the catalysts, manganese mono(bipyridyl) tricarbonyl,^16–18^ cobalt and iron porphyrin,^19–21^ cobalt tris(bipyridyl)^22^ and macrocycle,^23,24^ nickel cyclam,^25–27^ molybdenum and tungsten mono(bipyridyl) tetracarbonyl,^28^ rhodium bis(bipyridyl),^29,30^ palladium phosphine,^31–33^ rhenium mono(bipyridyl) tricarbonyl,^34–42^ osmium mono(bipyridyl) dicarbonyl,^43,44^ iridium poly(pyridyl) and dihydride pincer,^45,46^ ruthenium mono(bipyridyl) and bis(bipyridyl) dicarbonyl complexes^47–70^ have been investigated. Most of these yield CO and/or formate as the two-electron reduction products of CO_2_. Among the metal complex catalysts, ruthenium complexes (*e.g.*, [Ru(bpy)_2_(CO)_2_]^2+^) have actively been studied for the CO/HCOO^–^ selectivity. In the catalyses, the product selectivity depends on the reaction conditions: acidic conditions enhance CO production while basic conditions cause formate production.^51–56,69^ Photochemical reductions have mostly resulted in formate production^47–49,51–53,57–62,70^ while electrochemical reductions have achieved the selective formation of CO.^55,63–69^ The reaction mechanisms of Ru(ii) complexes have been proposed as shown in Scheme 1.^50–56,69^ The widely accepted mechanism of CO production is as follows: (1) the Ru(ii) complex accepts one electron to release CO, (2) the one-electron-reduced complex accepts another electron, and (3) the two-electron-reduced complex undergoes an electrophilic attack by CO_2_ along with protonation and dehydration to regenerate the starting Ru(ii) complex. For formate production, two mechanisms have been proposed so far. Tanaka and co-authors have proposed a mechanism in which the equilibrium between [Ru–C(O)OH]^(*n*+1)+^ and [Ru–CO]^(*n*+2)+^ governs the product selectivity between CO/HCOO^–^: the two-electron reduction of [Ru–C(O)OH]^(*n*+1)+^ causes formate production.^51–56,69^ This mechanism can explain well that formate is mainly produced under basic conditions where the equilibrium shifts to the hydroxycarbonyl complex. However, it is not fully accepted because the reaction requires a specific proton attack on the carbon atom of the hydroxycarbonyl group. Meyer *et al.* have proposed that formate is generated *via* insertion of CO_2_ into the Ru–H bond in [Ru–H]^*n*+^.^50^ The mechanism *via* the hydride complex is based on the experimental result that [Ru(bpy)_2_(CO)H]^0^ reacts with CO_2_ to yield [Ru(bpy)_2_(CO)(OC(O)H)]^0^, and is generally accepted as the mechanism of formate production in organometallic chemistry. However, this mechanism does not successfully elucidate why formate is selectively produced under less protic conditions and why dihydrogen originating from the hydride intermediate scarcely evolves when formate is produced, but does evolve with CO production. Even today with more than 20 years having passed since these mechanisms were proposed, consensus on the reaction mechanism of formate production has not yet been reached.

Recently, polymeric ruthenium mono(bipyridyl) dicarbonyl complexes (*e.g.*, [Ru(*L*)(CO)_2_]_*n*_ (*L* = bipyridyl derivatives)) have been utilized as reduction catalysts in artificial photosynthetic systems using semiconductors, which can utilize water as an electron donor.^60^ The system consists of TiO_2_ and InP modified with the ruthenium polymer. Photo-irradiation of the system induces electron transfer from the semiconductors to the ruthenium polymer, which catalyses CO_2_ reduction using the electrons to afford formate in a 10 mM NaHCO_3_ aqueous solution with CO_2_ bubbling. Polymeric ruthenium mono(bipyridyl) dicarbonyl complexes have been reported as catalysts in electrochemical CO_2_ reduction by Deronzier and Ziessel *et al.* ^63–65,67,68^ The selectivity of CO *vs.* formate production depends essentially on the substituents which are introduced at the 4,4' position of the bipyridyl ligand in aqueous solution.^64^ Ruthenium mono(bipyridyl) dicarbonyl polymers and their derivatives with electron-donating substituents give mainly CO as the reduction product at pH 6. The pH values of the solution and the electrolytes (*e.g.*, NaSO_4_ *vs.* LiClO_4_) used for the electrolyses moderately affect the selectivity. On the contrary, polymer complexes with electron-withdrawing substituents quantitatively yield HCOO^–^. This difference is explained by the electronic structures of the catalyst intermediates (the hydroxycarbonyl or formato complexes) formed during the electrocatalytic process. A similar tendency has been reported for electrocatalyses by derivatives of [Ru(bpy)_2_(CO)_2_]^2+^.^69^ Thus, results in the literature indicate that the reaction mechanisms and the product selectivity strongly depend on the reaction conditions. However, to the best of our knowledge, there is no report on the effects of catalyst concentration on product selectivity in CO_2_ reduction.

**Scheme 2:**
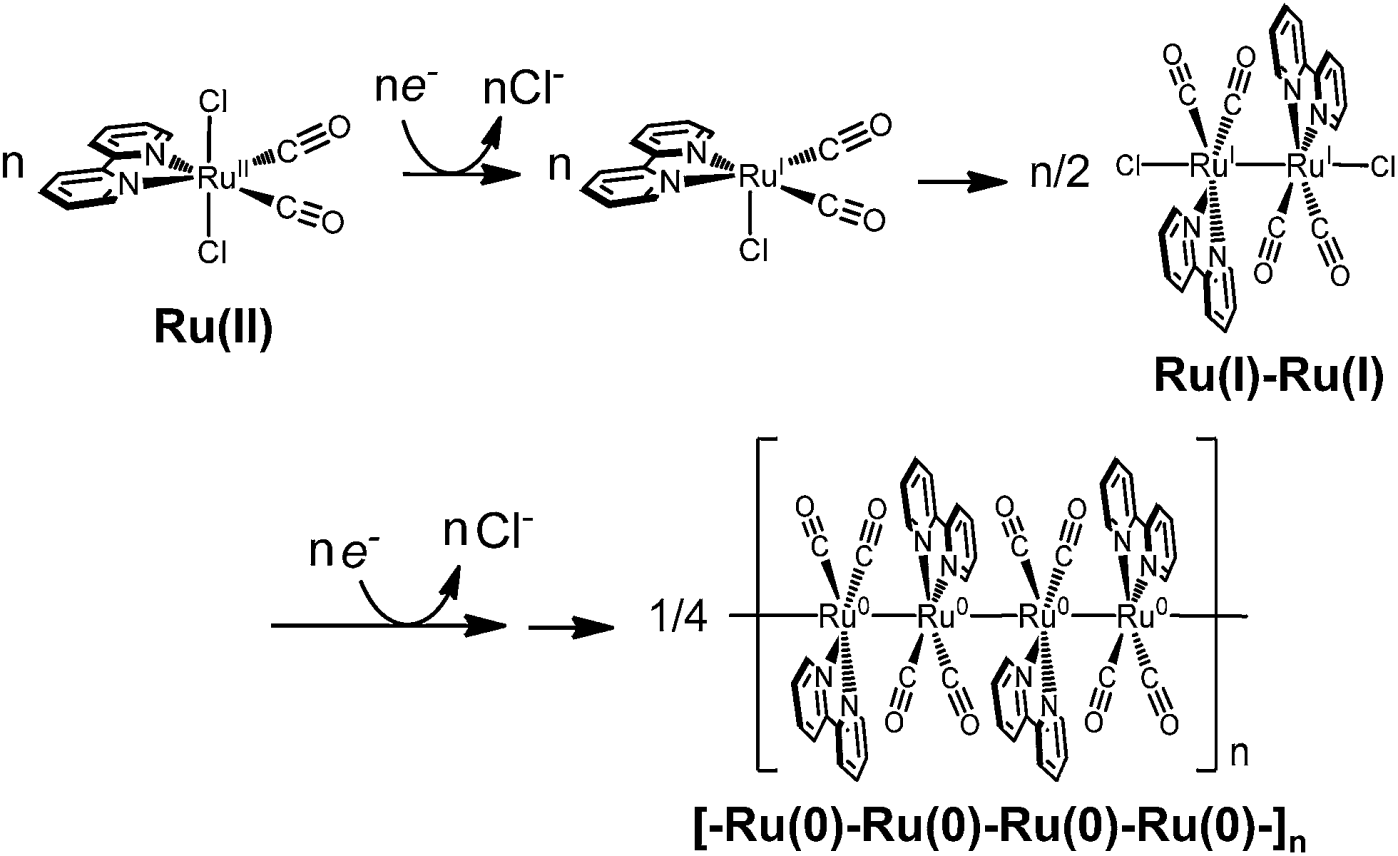
Formation of the polymeric ruthenium complex from *trans*(Cl)–Ru(bpy)(CO)_2_Cl_2_.

The polymeric ruthenium mono(bipyridyl) dicarbonyl complexes are obtained by electrochemical reductions of mono(bipyridyl) dicarbonyl dichloride complexes,^63–65,67,68,71,72^ as well as ruthenium bis(bipyridyl) dicarbonyl complexes.^66^ For instance, the formation process of [Ru(bpy)(CO)_2_]_*n*_ is shown in Scheme 2. The electrochemical reduction of Ru(bpy)(CO)_2_Cl_2_ initially dissociates the chloride ion to form a dimer, [Ru(bpy)(CO)_2_Cl]_2_, which has already been elucidated by Haukka *et al.* with X-ray crystallographic analysis.^73^ Further reduction of the ruthenium dimer causes dissociation of the chloride ions to give [Ru(bpy)(CO)_2_]_*n*_. The monomeric ruthenium complex, Ru(bpy)(CO)_2_Cl_2_, also shows catalytic activity for electrochemical CO_2_ reduction,^63,65,67,69^ but the complex tends to form an adherent film of the polymer on the electrode during electrochemical CO_2_ reduction, making it difficult to investigate the catalytic properties of the monomeric complex in detail. The photochemical CO_2_ reduction catalysed by Ru(bpy)(CO)_2_Cl_2_ has been reported in the presence of [Ru(bpy)_3_]^2+^ as the photosensitizer and triethanolamine (TEOA) as the electron donor,^70^ in which the reaction starts when [Ru(bpy)_3_]^2+^ absorbs visible light to induce electron transfer relay from TEOA to the catalyst. However, the system also causes the polymeric complex to form a black precipitate during the catalytic reaction, which probably inhibits light absorption and/or electron transfer from the photosensitizer.

We have very recently reported photochemical CO_2_ reduction catalysed by [Ru(bpy)_2_(CO)_2_]^2+^ in *N*,*N*-dimethylacetamide (DMA)/water containing [Ru(bpy)_3_]^2+^ and 1-benzyl-1,4-dihydronicotinamide (BNAH).^47^ DMA is used as an alternative solvent for *N*,*N*-dimethylformamide (DMF), which is the most frequently used solvent in photochemical CO_2_ reduction, but has been indicated to cause contamination of HCOO^–^ by hydrolysis.^74^ In the DMA/water systems using BNAH as the electron donor, the black precipitate scarcely formed, and the photocatalytic CO_2_ reduction proceeded smoothly. The products were CO and formate, which were confirmed as the CO_2_ reduction products by the ^13^C NMR experiments. We further showed that the oxidized form of BNAH was the BNA dimer, *e.g.*, 1,1′-dibenzyl-1,1′,4,4′-tetrahydro-4,4′-binicotinamide (4,4′-BNA_2_),^47,49^ indicating that the reduced species of the photosensitizer ([Ru(bpy)_3_]^+^), which was generated by the reductive quenching of the excited [Ru(bpy)_3_]^2+^ with BNAH, supplied the electrons to the catalyst.

**Fig. 1:**
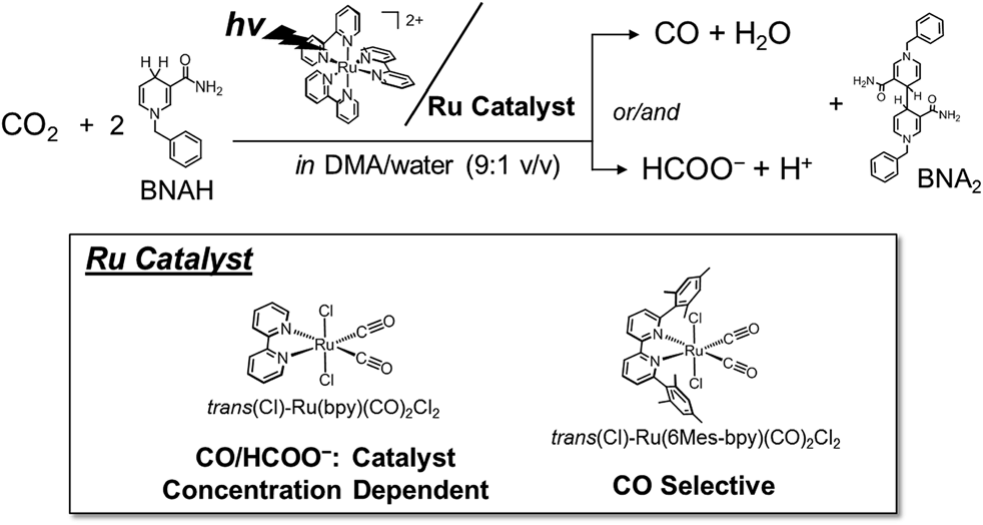
Photochemical CO_2_ reduction catalysed by *trans*(Cl)–Ru(bpy)(CO)_2_Cl_2_ or *trans*(Cl)–Ru(6Mes-bpy)(CO)_2_Cl_2_ in a DMA/water solution containing [Ru(bpy)_3_]^2+^ and BNAH as the photosensitizer and the electron donor, respectively.

In this work, we have investigated photochemical CO_2_ reduction catalysed by *trans*(Cl)–Ru(bpy)(CO)_2_Cl_2_ in a DMA/water solution containing [Ru(bpy)_3_]^2+^ and BNAH (Fig. 1). We have unexpectedly discovered that the concentration of *trans*(Cl)–Ru(bpy)(CO)_2_Cl_2_ affects the product selectivity (CO/HCOO^–^). The mechanisms reported so far^48,50–56,64,69,70^ cannot explain the phenomenon. This motivated us to reconsider the reaction mechanism of photochemical CO_2_ reduction. We propose a new reaction mechanism involving a Ru(i)–Ru(i) dimer, which catalyses CO_2_ reduction to selectively produce formate, because HCOO^–^ production becomes dominant under low-intensity light, in which the Ru–Ru bond tends to form. The photochemical CO_2_ reduction catalysed by [Ru(bpy)(CO)_2_Cl]_2_ (0.05 mM) shows similar time–course profiles for the production of CO and HCOO^–^ to those of *trans*(Cl)–Ru(bpy)(CO)_2_Cl_2_ (0.10 mM), indicating that the immediate dissociation of the dimer occurs to regenerate the monomeric complex during the CO_2_ reduction. We carried out kinetic analyses based on the new reaction mechanism, and the simulation curve reproduced the concentration dependence of the product selectivity (CO/HCOO^–^) well. In order to further verify the proposed mechanism, we synthesized *trans*(Cl)–Ru(6Mes-bpy)(CO)_2_Cl_2_ (Fig. 1, 6Mes-bpy: 6,6′-dimesityl-2,2′-bipyridine) which cannot form a dimer due to the bulky substituents at 6,6′-positions in 2,2′-bipyridine. The photochemical CO_2_ reduction catalysed by *trans*(Cl)–Ru(6Mes-bpy)(CO)_2_Cl_2_ selectively produced CO, strongly supporting our proposed mechanism for formate production.

## Experimental section

### General procedure

[Ru(bpy)(CO)_2_Cl]_2_, [Ru(CO)_2_Cl_2_]_*n*_, *trans*(Cl)–Ru(bpy)(CO)_2_Cl_2_, [Ru(bpy)_3_](PF_6_)_2_, [Ru(4dmbpy)_3_](PF_6_)_2_ (4dmbpy = 4,4′-dimethyl-2,2′-bipyridine), 6,6′-dimesityl-2,2′-bipyridine (6Mes-bpy), and BNAH were prepared according to the literature.^73,75–78^ DMA (Wako, dehydrate) was used as supplied. High-purity water (resistivity: 18.2 MΩ cm) was obtained from an ultrapure water system (RFU424TA, Advantec). Cyclic voltammograms (CVs) and differential pulse voltammograms (DPVs) were obtained using a Bio-Logic VSP Potentiostat using EC-Lab software. As the electrodes, a BAS glassy-carbon working electrode, a BAS Pt counter electrode, and a BAS RE-7 (Ag/AgNO_3_ 0.01 M in acetonitrile) reference electrode were used. Absorption spectra in the spectroelectrochemical experiments were obtained on an ALS SEC2000 using an electrochemical cell of 1 mm path length incorporating the three-electrode system.

### Synthesis of *trans*(Cl)–Ru(6Mes-bpy)(CO)_2_Cl_2_

In a 30 mL flask equipped with a reflux condenser were placed [Ru(CO)_2_Cl_2_]_*n*_ (30 mg), 6,6′-dimesityl-2,2′-bipyridine (6Mes-bpy; 51 mg, 0.13 mmol) and ethanol (6 mL) under an argon atmosphere, and the solution was refluxed for 18 h. As the reaction proceeded, the starting solution became a white suspension. The precipitate was filtrated and washed with ethanol. The solid was recrystallized from CHCl_3_–ether to afford pale yellow crystals (45 mg, 56%): ^1^H NMR (400 MHz, CDCl_3_) *δ* 8.26 (d, *J* = 8.0 Hz, 2H), 8.09 (dd, *J* = 8.0 and 7.6 Hz, 2H), 7.45 (d, *J* = 7.6 Hz, 2H), 6.97 (s, 4H), 2.33 (s, 6H), 2.16 (s, 12H); FTIR (KBr) ν_CO_/cm^–1^ 1986, 2051. Anal. calcd (%) for C_30_H_28_Cl_2_N_2_O_2_Ru: C, 58.07; H, 4.55; N, 4.51. Found: C, 58.11; H, 4.76; N, 4.65.

### Photocatalytic CO_2_ reduction

Solutions (5 mL) of the catalyst (*trans*(Cl)–Ru(bpy)(CO)_2_Cl_2_ or *trans*(Cl)–Ru(6Mes-bpy)(CO)_2_Cl_2_), [Ru(bpy)_3_](PF_6_)_2_ and BNAH in Ar-saturated DMA/water were placed in quartz tubes (23 mL volume, i.d. = 14 mm). The solutions were bubbled through septum caps with CO_2_ gas for 20 min, and then were irradiated using a 400 W high-pressure mercury lamp at *λ* > 400 nm (Riko Kagaku, L-39 cutoff filter) in a carousel irradiation apparatus (Riko Kagaku, RH400-10W). The reaction temperature was maintained at 298 ± 3 K by using a water bath. The gaseous products (CO and H_2_) were analyzed with GC, and formate was also quantified with GC by acidifying formate to formic acid.^47^

### Quenching experiments

Emission from the excited state of [Ru(4dmbpy)_3_](PF_6_)_2_ in the Ar-saturated DMA/water solution was recorded on a Hitachi F-4500 spectrometer (*λ*_ex_ = 453 nm) in the absence and in the presence of the quencher, BNAH. The Stern–Volmer relationship (eqn (1)) was obtained from the plots of the relative emission intensity (*I*_0_/*I*) *versus* the concentration of the quencher (Q: BNAH):

1 *I*_0_/*I* = 1 + *K*_SV_[*Q*] = 1 + *k*_q_*τ*[*Q*]

where *I*_0_ and *I* represent the intensity at 628 nm in the absence and the presence of the quencher, respectively, and *K*_sv_, *k*_q_, *τ* are the Stern–Volmer constant, the quenching rate constant, and the emission lifetime, respectively.

### Light intensity dependence

In square quartz cells (*l* = 1.0 cm) were placed DMA/water (9 : 1 v/v, 3 mL) solutions containing the Ru catalyst, [Ru(bpy)_3_](PF_6_)_2_ and BNAH, and CO_2_ was bubbled through the septum caps for at least 30 min before measurement. The solutions were irradiated using a 500 W superhigh-pressure mercury lamp (Ushio, USH-500D) through a Toshiba Y-43 glass filter (*λ* > 400 nm) with and without neutral-density (ND) filters. The absorption spectra of the solutions were measured with a Shimadzu MultiSpec-1500 Spectrometer. The gaseous products (CO and H_2_) were analysed with GC, and formate was also quantified with GC by acidifying formate to formic acid.^47^

## Results and discussion

### Photochemical CO_2_ reduction catalysed by *trans*(Cl)–Ru(bpy)(CO)_2_Cl_2_

The photocatalytic CO_2_ reductions were carried out in DMA/water (9 : 1 v/v) solutions containing *trans*(Cl)–Ru(bpy)(CO)_2_Cl_2_ (the catalyst), [Ru(bpy)_3_]^2+^ (the photosensitizer) and BNAH (the electron donor) under visible light irradiation (*λ* > 400 nm), where [Ru(bpy)_3_]^2+^ was selectively excited and reductively quenched by BNAH to yield the reduced species of the photosensitizer, [Ru(bpy)_3_]^+^.^47^ The 10 vol% water content was selected for the reaction solvent because this water ratio gave the highest amount of the reduction products. The water in the reaction solution plays an important role as the transporter of the protons for the CO_2_ reduction, but higher contents of water decrease the quenching efficiency of BNAH toward [Ru(bpy)_3_]^2+*^.^47^ The reduction potential of *trans*(Cl)–Ru(bpy)(CO)_2_Cl_2_, which was estimated to be –1.51 V *vs.* Ag/Ag^+^ in DMA/water (9 : 1 v/v) with the use of the differential pulse voltammetry (Fig. S1 in ESI†), indicates that electron transfer can occur thermodynamically from the reduced photosensitizer ([Ru(bpy)_3_]^2+/+^: –1.68 V *vs.* Ag/Ag^+^)^47^ to *trans*(Cl)–Ru(bpy)(CO)_2_Cl_2_.

**Fig. 2:**
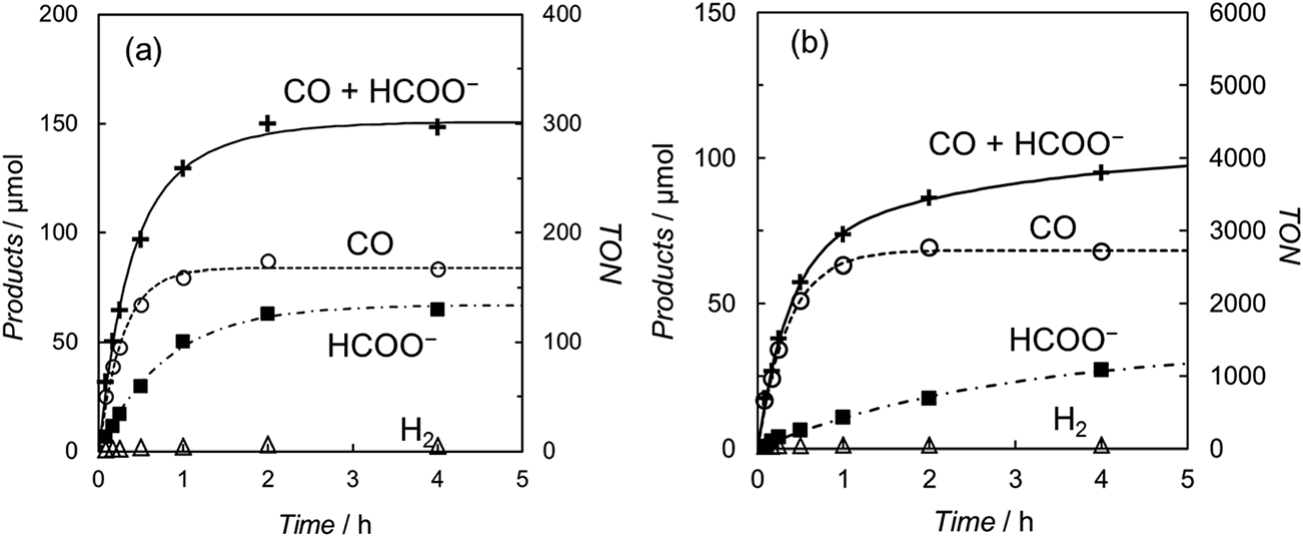
Photo-irradiation time dependence of the products in a CO_2_-saturated DMA/water (9 : 1 v/v) solution containing (a) 0.1 mM and (b) 5.0 μM of *trans*(Cl)–Ru(bpy)(CO)_2_Cl_2_, [Ru(bpy)_3_](PF_6_)_2_ (0.50 mM) and BNAH (0.10 M): CO (○), HCOO^–^ (■), H_2_ (Δ) and CO + HCOO^–^ (+).

Fig. 2 shows the time-courses of the products in the photochemical CO_2_ reduction in the CO_2_-saturated DMA/water (9 : 1 v/v) solution containing *trans*(Cl)–Ru(bpy)(CO)_2_Cl_2_ ((a) 0.10 mM and (b) 5.0 μM), [Ru(bpy)_3_]^2+^ (0.50 mM) and BNAH (0.10 M). The profiles show that CO and formate are selectively yielded, with scarce accompanying H_2_ evolution even in the aqueous solutions, suggesting that the reduced catalyst reacts much more favourably with CO_2_ than H^+^. The turnover number (TON) for the total amount of CO and formate was *ca.* 300 at 0.10 mM of the catalyst after photo-irradiation for 4 h. When 5.0 μM of *trans*(Cl)–Ru(bpy)(CO)_2_Cl_2_ was used, the TON was dramatically improved to *ca.* 4000, because there was no superfluous catalyst at the lower concentration. However, the rate of the formation of the products becomes slow over 2 hours. In our previous work, we reported that these effects are mainly attributable to the decrease of BNAH and the increase of BNA_2_.^47^ The latter depresses the photochemical CO_2_ reduction because BNA_2_ reductively quenches the excited state of [Ru(bpy)_3_]^2+^ faster than BNAH, but the back electron transfer is much more efficient.

**Fig. 3:**
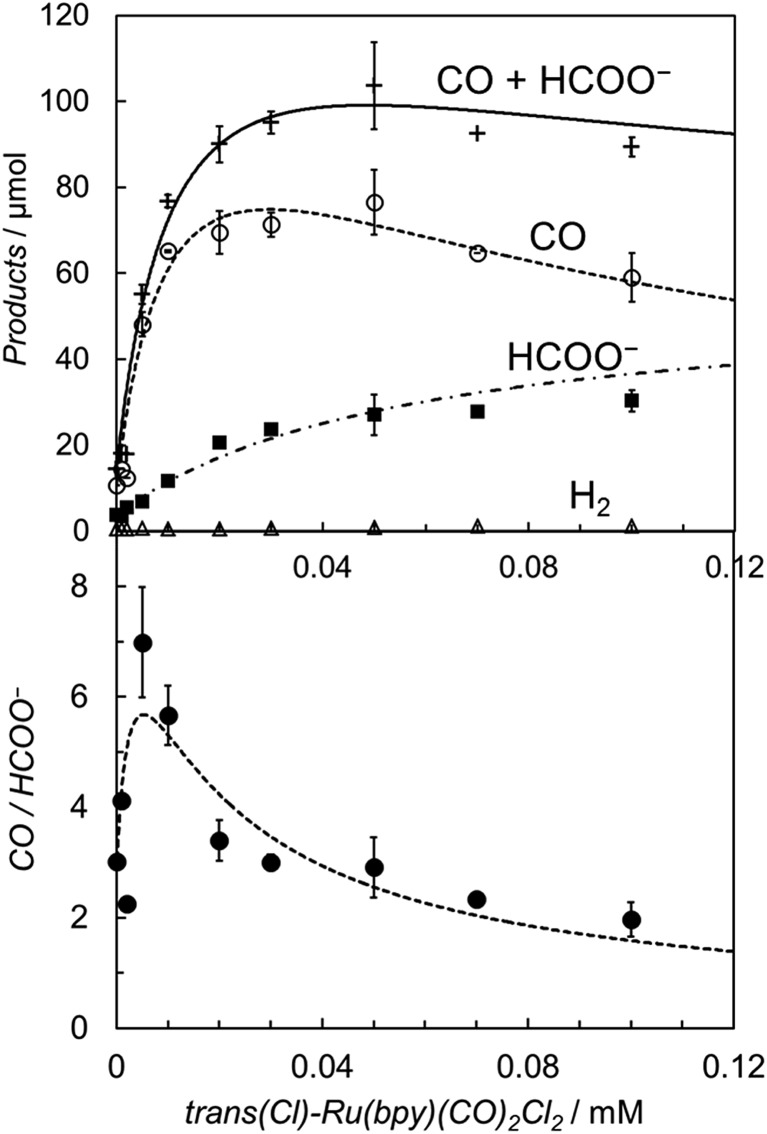
(Top) Plots of the amounts of the reduction products after 30 min of photo-irradiation (400 W Hg lamp, *λ* > 400 nm) *versus* the concentration of *trans*(Cl)–Ru(bpy)(CO)_2_Cl_2_ in CO_2_-saturated DMA/water (9 : 1 v/v) in the presence of [Ru(bpy)_3_](PF_6_)_2_ (0.50 mM) and BNAH (0.10 M): CO (○), HCOO^–^ (■), H_2_ (Δ) and CO + HCOO^–^ (+). (Bottom) Plots of the CO/HCOO^–^ ratio *versus* the concentration of *trans*(Cl)–Ru(bpy)(CO)_2_Cl_2_. The curves represent the theoretical fittings based on the kinetic analyses (see eqn (3) and (5)).

It is worth noting that the product ratio of CO/HCOO^–^ in Fig. 2 is notably higher at 5.0 μM of *trans*(Cl)–Ru(bpy)(CO)_2_Cl_2_ than that at 0.1 mM. For example, the ratio of CO/HCOO^–^ is approximately 2 at 0.1 mM of the catalyst but *ca.* 7 at 5.0 μM after 30 min of photo-irradiation. Fig. 3 shows the dependence of the amounts of CO and formate and the product ratio of CO/HCOO^–^ on the catalyst concentration after 30 min of photo-irradiation, which reflects the initial reaction rates of the CO_2_ reduction. In Fig. 3 (bottom), the initially increased product ratio of CO/HCOO^–^ is lowered by increasing the catalyst concentration. The unexpected profile of the product ratio comes from the differing behaviours of the initial production rates of CO and formate: the rate of CO formation increases as the catalyst concentration increases up to 20–30 μM, then decreases as the concentration increases above 30 μM, while the rate of formate production continues to increase as the catalyst concentration increases. In Fig. 3 (bottom), the increase in the product ratio with increasing catalyst concentration from 0 to 5.0 μM comes from the contribution of the blank products, which are detected even in the absence of *trans*(Cl)–Ru(bpy)(CO)_2_Cl_2_. It is known that [Ru(bpy)_3_]^2+^ releases the bipyridyl ligand by photo-labilization to provide catalytically active species, resulting in the blank products.^57,70^ The amounts of the blank products are 11 and 4 μmol for CO and formate, respectively, and the blank product ratio of CO/HCOO^–^ is *ca.* 3. Thus, if the blank products caused by [Ru(bpy)_3_]^2+^ were excluded, the selectivity of CO would continue to increase with decreasing catalyst concentration.

### Changes in the electronic absorption spectra during the photo-irradiation and light intensity dependence of the product selectivity

**Fig. 4:**
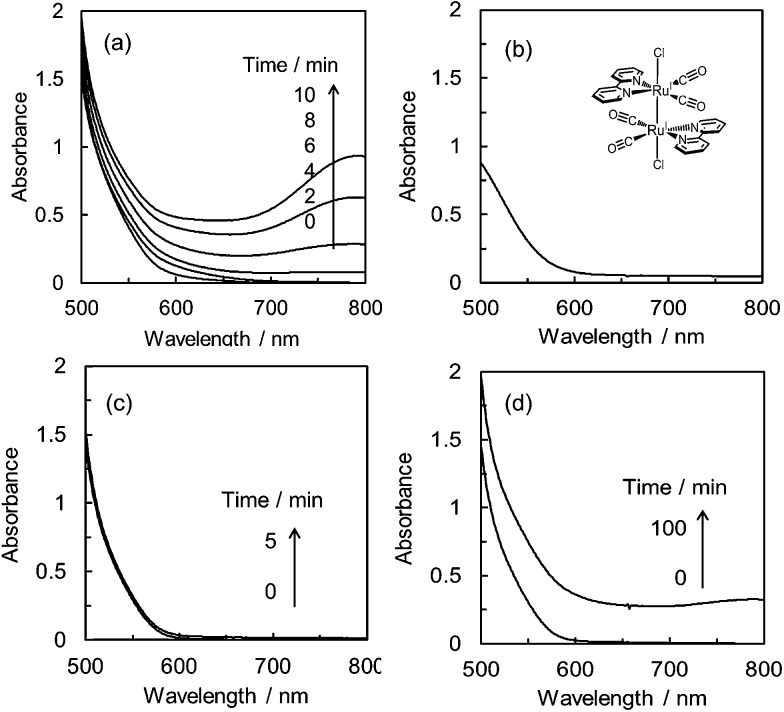
(a) Absorption spectra of an Ar-saturated DMA/water (9 : 1 v/v) solution containing *trans*(Cl)–Ru(bpy)(CO)_2_Cl_2_ (0.20 mM), [Ru(bpy)_3_](PF_6_)_2_ (0.50 mM) and BNAH (0.10 M) during photo-irradiation with *λ* > 400 nm light with an intensity of 7.5 × 10^–7^ einstein s^–1^. (b) Absorption spectrum of [Ru(bpy)(CO)_2_Cl]_2_ (0.40 mM) in DMA/water (9 : 1 v/v). (c) Absorption spectra of CO_2_-saturated DMA/water (9 : 1 v/v) solutions containing *trans*(Cl)–Ru(bpy)(CO)_2_Cl_2_ (0.10 mM), [Ru(bpy)_3_](PF_6_)_2_ (0.50 mM) and BNAH (0.10 M) by photo-irradiation with *λ* > 400 nm light of 7.5 × 10^–7^ einstein s^–1^ and (d) 3.8 × 10^–8^ einstein s^–1^ (total incident light: 2.3 × 10^–4^ einstein).

As shown in Fig. 3, the product selectivity of CO/HCOO^–^ was affected by the catalyst concentration. The behaviour led us to consider that an association of the catalyst occurs during the CO_2_ reduction. The photo-irradiation of an Ar-saturated DMA/water solution containing *trans*(Cl)–Ru(bpy)(CO)_2_Cl_2_ (0.20 mM), [Ru(bpy)_3_]^2+^ and BNAH produced H_2_ instead of CO and formate. During the photo-irradiation, the solution colour changed from orange to dark red. The spectra of the Ar-saturated reaction solution showed the appearance of a characteristic broad peak at 700–800 nm (Fig. 4a), indicating that the polymeric ruthenium complex, [Ru(bpy)(CO)_2_]_*n*_, formed by the reduction of *trans*(Cl)–Ru(bpy)(CO)_2_Cl_2_.^49,79,80^ On the other hand, photo-irradiation (400 W high-pressure mercury lamp without the ND filter, *λ* > 400 nm) under a CO_2_ atmosphere showed no colour change of the solution, suggesting that the reduced catalyst was oxidized by coordination with CO_2_ to suppress the formation of the polymeric complex. It has been reported that *trans*(Cl)–Ru(bpy)(CO)_2_Cl_2_ is electrochemically reduced to form the polymeric ruthenium complex *via* the Ru(i)–Ru(i) dimer (Scheme 2).^71,72^ As the absorption band of the dimer overlaps with that of [Ru(bpy)_3_]^2+^ (Fig. 4b), the formation of the dimer could not be observed using the absorption spectra. Thus, if the product selectivity of CO/HCOO^–^ is related to an association of the catalysts, and changes to the absorption spectrum of the reaction solution are not observed during the photo-irradiation, the associated species might be the ruthenium dimeric complex.

Fig. 4c and d show the spectra during photochemical CO_2_ reduction under high- and low-intensity light (500 W superhigh-pressure mercury lamp without and with the ND filters, *λ* > 400 nm). The irradiation time was adjusted for the total incident light to be 2.3 × 10^–4^ einstein. While no spectral change is observed in Fig. 4c, a polymeric absorption at 700–800 nm appears in Fig. 4d. This indicates that the Ru–Ru bond tends to form under the lower-intensity light.

**Fig. 5:**
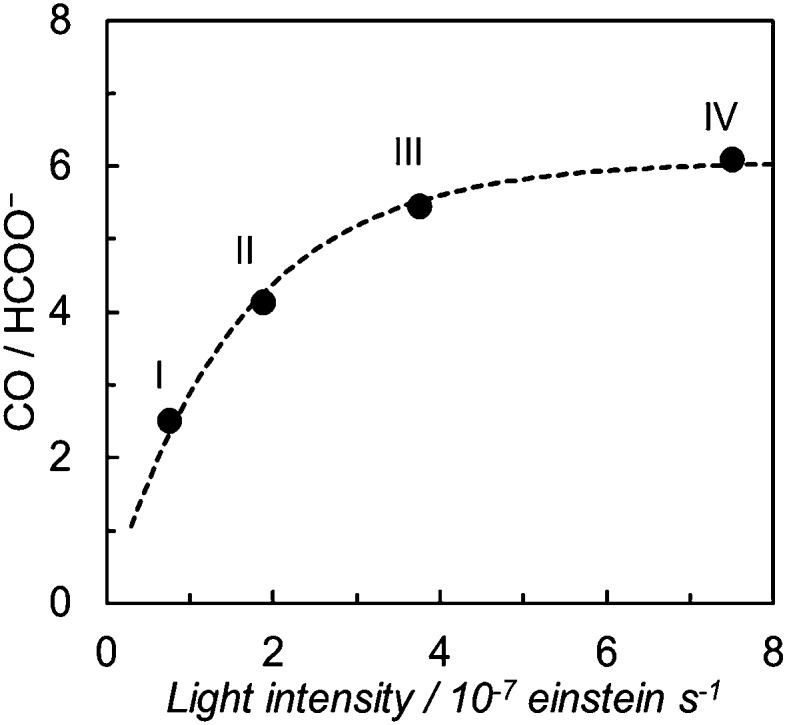
Light intensity dependence of the product ratio of CO/HCOO^–^ in DMA/water (9 : 1 v/v) solutions containing *trans*(Cl)–Ru(bpy)(CO)_2_Cl_2_ (20 μM), [Ru(bpy)_3_](PF_6_)_2_ (0.50 mM) and BNAH (0.10 M) during photo-irradiation with *λ* > 400 nm light. The photo-irradiation times were (I) 50 min, (II) 20 min, (III) 10 min and (IV) 5 min (total incident light: 2.3 × 10^–4^ einstein).

We have further investigated the light intensity dependence of the product selectivity in the photochemical CO_2_ reduction. Fig. 5 shows the light intensity dependence of the product ratio at a dilute concentration of *trans*(Cl)–Ru(bpy)(CO)_2_Cl_2_ (20 μM). The ratio of CO/HCOO^–^ decreases with reduction of the light intensity, that is, the formation of HCOO^–^ becomes dominant at lower light intensity. Since the concentration of 20 μM was selected to prevent the formation of the polymer that caused a dramatic decrease of the effective catalyst concentration in the solution, we could exclude the possibility that the polymeric species contributed to the product selectivity. Considering that the low-intensity light induces the Ru–Ru bond formation, the most plausible key intermediate for forming HCOO^–^ would be the ruthenium dimer.

### Mechanistic insight into product selectivity

**Scheme 3:**
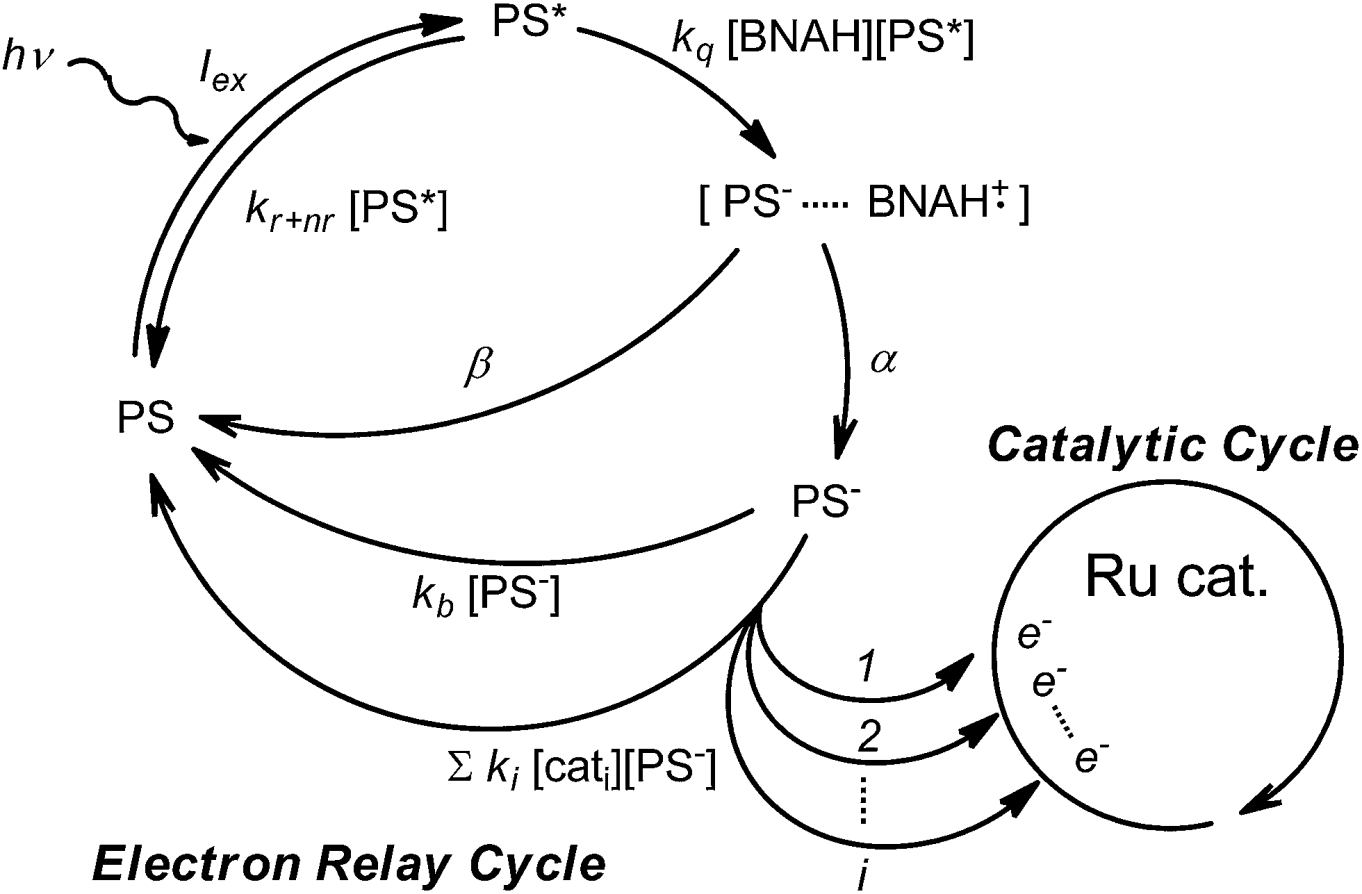
The electron relay cycle and the catalytic cycle in the photochemical CO_2_ reduction.

The photochemical CO_2_ reduction system consists of two parts: an electron relay cycle and a catalytic cycle (Scheme 3). In the electron relay cycle, the excited state of the photosensitizer (PS*) and BNAH form the encounter complex, where electron transfer from BNAH to PS* occurs to produce the charge-separated state ([PS^–^···BNAH·^+^]).^81,82^ Dissociation of the encounter complex gives the free reduced photosensitizer (PS^–^), which could supply electrons to the catalyst. In Scheme 3, *I*_ex_ is the rate of incident photons, *k*_q_ is the quenching rate constant by BNAH, *k*_*r+nr*_ is the sum of the radiative and non-radiative rate constants of the PS*, *α* is the cage escape efficiency after the electron transfer from BNAH to the PS*,^82^ *β* is the fraction of back-electron transfer in the solvent cage (*β* = 1 – *α*), *k*_b_ is the quenching rate constant of the PS^–^, *k*_*i*_ is the electron transfer rate constant from the PS^–^ to the *i*th form of the catalyst, and [cat_*i*_] is the concentration of the *i*th form of the catalyst. In the initial stage of the reaction, the quenching by BNA_2_ can be ignored.^47^ The steady-state concentration of the PS^–^ is evaluated as a function of the concentration of the catalyst (eqn (2)) by applying the steady state approximation to Scheme 3:2
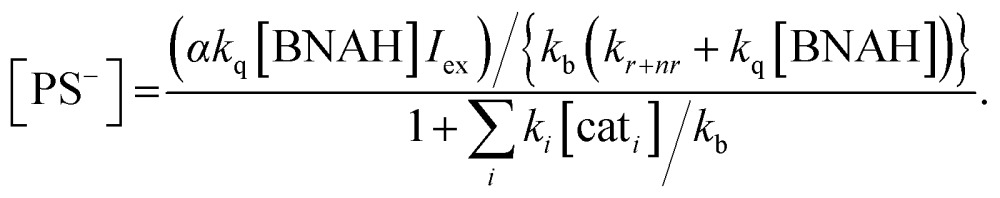



According to eqn (2), when the light intensity and the concentration of BNAH are constant, the steady-state concentration of the PS^–^ is affected by the catalyst concentration: [PS^–^] decreases as the catalyst concentration increases.

**Scheme 4:**
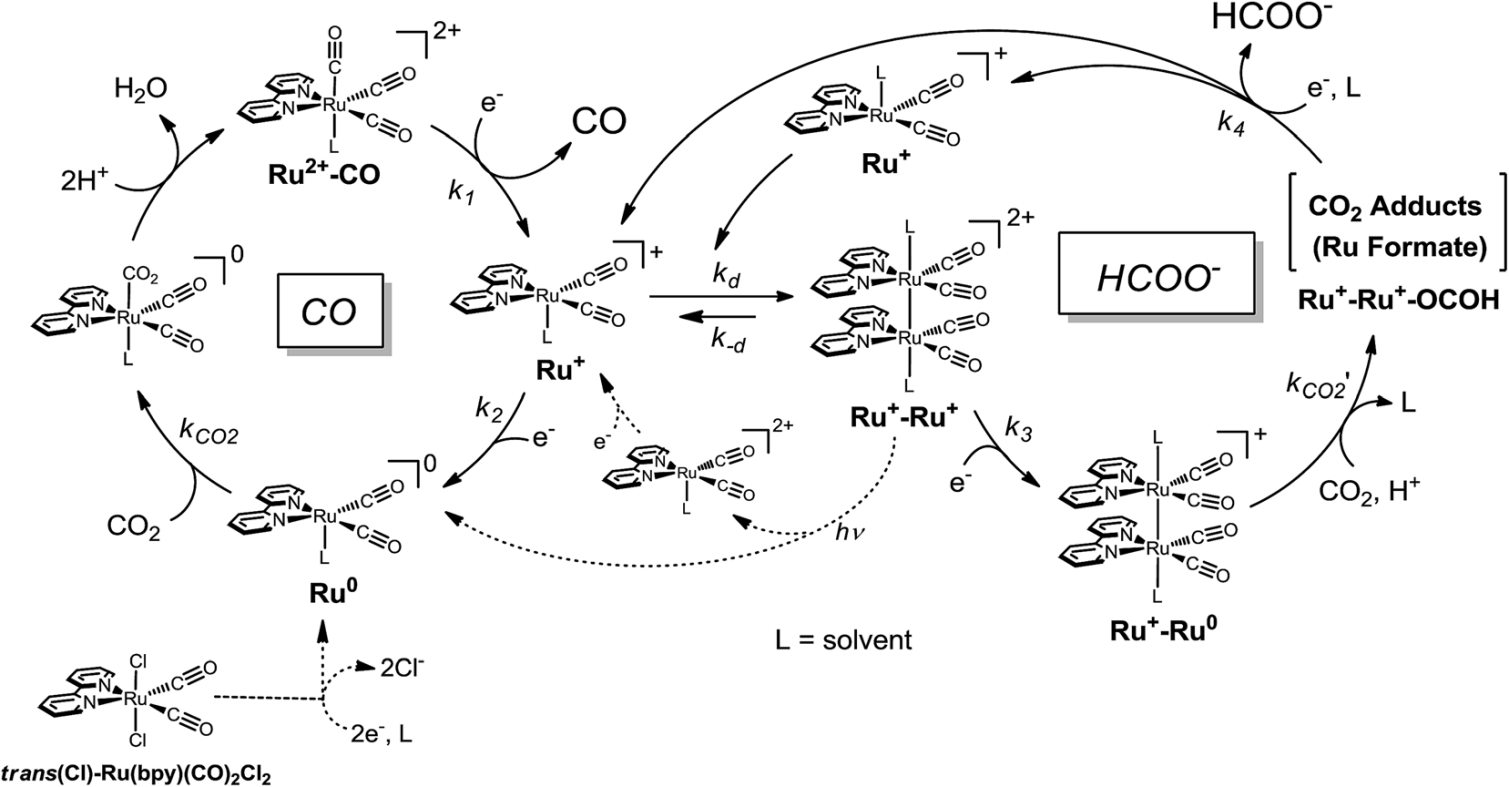
Plausible mechanism for CO_2_ reduction.

In the catalytic cycle, we have assumed that the dimeric complex produces formate while the monomeric complex produces CO through reaction with CO_2_ (Scheme 4). It has been reported that the one-electron-reduced catalyst (Ru^+^) does not react with CO_2_ (ref. 48–56, 69, 70 and 83) but instead forms a dimer Ru^+^–Ru^+^.^71,72^ Therefore the resting state in the catalytic cycle would be the Ru^+^ species. In the low concentration region of the catalyst, it is possible for the catalyst to accept two electrons smoothly from the PS^–^. On the other hand, in the high concentration region, the amount of the PS^–^ would not be enough for the catalyst to receive two electrons smoothly. In particular, in the photochemical reaction, electron transfer to the catalyst hardly occurs, and accordingly the Ru^+^ remains unreacted.

The valence of the ruthenium centre in the dimer is possibly changed by the reaction with CO_2_ and H^+^. When the Ru(ii) valence state forms, the Ru–Ru bond would dissociate into the monomeric species. In order to confirm the cleavage of the Ru–Ru bond, we examined the photocatalytic CO_2_ reduction by [Ru(bpy)(CO)_2_Cl]_2_, which was separately prepared.^73^ According to the reduction potential of [Ru(bpy)(CO)_2_Cl]_2_, which is estimated to be *ca.* –1.6 V *vs.* Ag/Ag^+^ in DMA/water (9 : 1 v/v) (Fig. S2 in ESI†), electron transfer can thermodynamically occur from the reduced photosensitizer to the dimer. The reaction catalysed by 0.05 mM of [Ru(bpy)(CO)_2_Cl]_2_ (0.10 mM of the Ru unit) showed very similar time–course profiles of CO and HCOO^–^ to those of 0.10 mM of *trans*(Cl)–Ru(bpy)(CO)_2_Cl_2_ (Fig. S3 in ESI†). This result supports formate production being accompanied with the cleavage of the Ru–Ru bond to regenerate the monomeric species. We carried out the photo-irradiation of a concentrated solution of [Ru(bpy)(CO)_2_Cl]_2_ (0.40 mM) in Ar-saturated DMA/water without either the photosensitizer or the electron donor. The direct photo-irradiation of the dimer results in the appearance of a broad absorption corresponding to the polymeric species, suggesting that disproportionation of the Ru(i)–Ru(i) dimer occurs (Scheme 4). The disproportionation of the dimer has been proposed in the isomerization from *trans*(Cl) to *cis*(Cl)–Ru(bpy)(CO)_2_Cl_2_, which is induced by the addition of NaBH_4_.^84^ However, in the reaction conditions in the presence of [Ru(bpy)_3_]^2+^ (0.50 mM) as the photosensitizer, direct photo-excitation of the dimer scarcely occurs because most of the light is absorbed by the photosensitizer when the concentration of the dimer is low. In addition, we have observed that [Ru(bpy)(CO)_2_Cl]_2_ is stable against CO_2_ in DMA/water in the dark. Thus, it is thought that formate production would start with the electrical reduction of Ru^+^–Ru^+^.

The relationship between the concentration of the catalyst and the initial rates for the formation of CO and formate was evaluated by applying the steady state approximation to Scheme 4 and using eqn (2). The equations for CO and formate are expressed as the following eqn (3) and (4), respectively (See ESI†):3
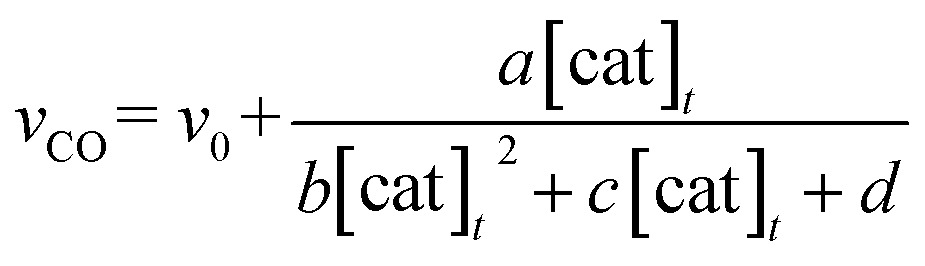

4
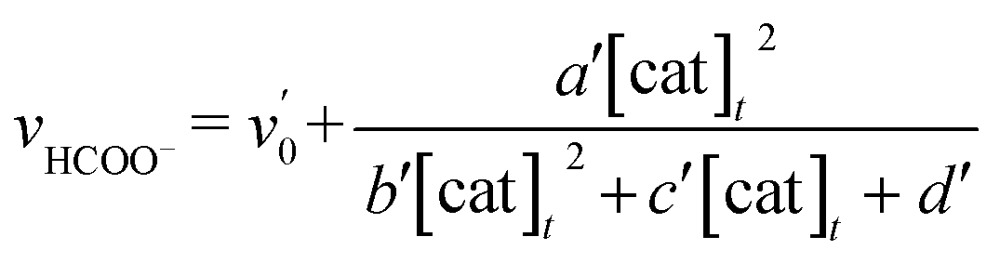
where *v*_CO_ and *v*_HCOO^–^_ are the formation rates of CO and formate, which can be calculated by dividing the concentration in mol of the products after photo-irradiation for 30 min by the volume of the reaction solution (5.0 mL) and the time (1800 s); [cat]_*t*_ is the initial concentration of *trans*(Cl)–Ru(bpy)(CO)_2_Cl_2_; *γ* is a proportional constant particular to the catalyst, *γ* = [Ru^+^]/[cat]_*t*_; *v*_0_ and *v*_0_′ are the blank formation rates caused by [Ru(bpy)_3_]^2+^; *a*, *b*, *c*, *d*, *a*′, *b*′, *c*′ and *d*′ are the constant values as expressed by the following: *a* = (*k*_2_ *k*_–d_ *α k*_q_ [BNAH] *I*_ex_ *γ*)/(*k*_*n*+*nr*_ + *k*_q_ [BNAH]), *b* = *b*′ = 2 *k*_d_ *k*_3_ *γ* ^2^, *c* = *c*′ = 2 *k*_–d_ *k*_2_ *γ*, *d* = *k*_b_ *k*_–d_ + (*k*_3_ *α k*_q_ [BNAH] *I*_ex_)/(*k*_*n*+*nr*_ + *k*_q_ [BNAH]), *a*′ = (*k*_3_ *k*_d_ *α k*_q_ [BNAH] *I*_ex_ *γ* ^2^)/(*k*_*n+nr*_ + *k*_q_ [BNAH]), *d*′ = *k*_b_ *k*_–d_. The value of *γ* is related to *k*_CO_2__ and *k*_CO_2__′, and a higher value of *γ* would indicate a higher reaction rate of the two-electron-reduced catalyst with CO_2_ and H^+^.

In Fig. 3 top, curve fitting according to eqn (3) gives the parameters: *v*_0_ = 1.2 × 10^–6^ M s^–1^, *a*/*d* = 1.1 s^–1^, *b*/*d* = 1.1 × 10^9^ M^–2^, *c*/*d* = 9.1 × 10^4^ M^–1^. The simulation curve well reproduces the experimental behaviour, where the rate increases as the catalyst concentration increases up to 20–30 μM then decreases as the concentration increases above 30 μM. Eqn (4) can be simplified when the *d*′ term is negligible (Fig. S9 in ESI†):5
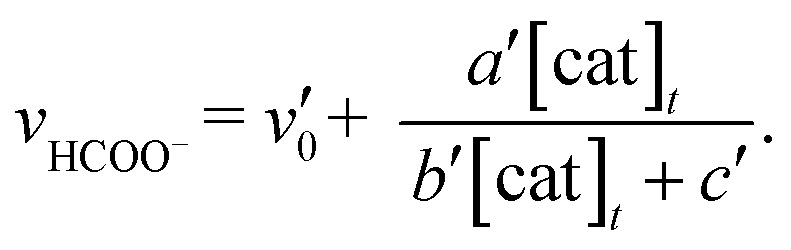



**Fig. 6:**
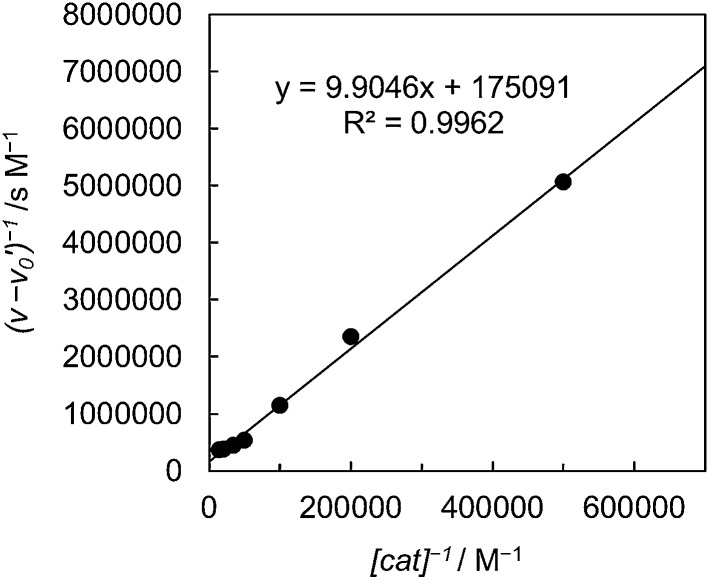
Double-reciprocal plots of the rate of the formate production *versus* the concentration of *trans*(Cl)–Ru(bpy)(CO)_2_Cl_2_.

By assuming *v*_0_′ = 4.2 × 10^–7^ M s^–1^, the double-reciprocal plots of the rate of formate production *versus* the concentration of *trans*(Cl)–Ru(bpy)(CO)_2_Cl_2_ give *a*′/*c*′ = 0.10 s^–1^ and *b*′/*c*′ = 1.8 × 10^4^ M^–1^ (Fig. 6). These simulation curves based on eqn (3) and (5) agree well with the experimental plots in Fig. 3 (top), and also reproduce the results for the selectivity in Fig. 3 (bottom).

In eqn (3)–(5), the following relationships should be satisfied; *b*/*c* = *b*′/*c*′ and *a*/*c* = *a*′/*b*′. From eqn (3) and (5), *b*/*c* and *b*′/*c*′ are estimated to be 1.3 × 10^4^ M^–1^ and 1.8 × 10^4^ M^–1^, and *a*/*c* and *a*′/*b*′ to be 1.2 × 10^–5^ M s^–1^ and 0.6 × 10^–5^ M s^–1^, respectively. The results of the curve fittings satisfy the theoretical requirements. Furthermore, *a*/*c* and *a*′/*b*′ are expressed as eqn (6) and the value can be estimated using the values *k*_q_ = 2.6 × 10^8^ M^–1^ s^–1^, *k*_*r*+*nr*_ = 1.2 × 10^6^ s^–1^ and *αI*_ex_ = 3.3 × 10^–5^ M s^–1^, which are obtained from the Stern–Volmer plot, the emission lifetime of the excited [Ru(bpy)_3_]^2+^ and the simulation curve of the decrease of BNAH, respectively.^47^
6
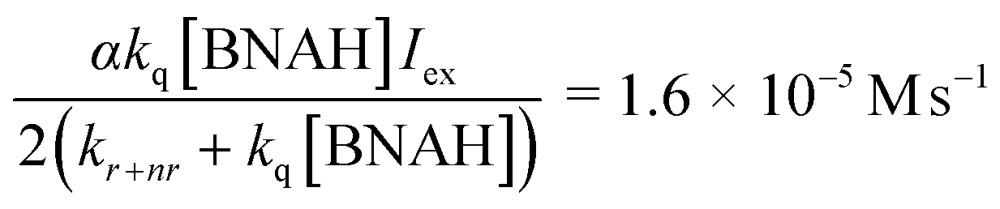



This value is consistent with those of *a*/*c* and *a*′/*b*′ estimated from eqn (3) and (5), indicating that the kinetic analyses strongly support the proposed mechanism.

### Photochemical CO_2_ reduction using [Ru(4dmbpy)_3_]^2+^ as photosensitizer

As the photosensitizer in the photochemical CO_2_ reduction, we used [Ru(bpy)_3_]^2+^, whose first reduction potential is –1.68 V *vs.* Ag/Ag^+^ in DMA/water (9 : 1 v/v).^47^ In order to investigate the effect of the photosensitizer on the product selectivity, we used [Ru(4dmbpy)_3_]^2+^ (4dmbpy = 4,4′-dimethyl-2,2′-bipyridine) instead of [Ru(bpy)_3_]^2+^. [Ru(4dmbpy)_3_]^2+^ has a more negative reduction potential of –1.77 V *vs.* Ag/Ag^+^ (Fig. S4†) than that of [Ru(bpy)_3_]^2+^, indicating that [Ru(4dmbpy)_3_]^2+^ works as a powerful reductant after the photo-induced electron transfer from BNAH is completed. However, the more negative potential of [Ru(4dmbpy)_3_]^2+^ makes the quenching rate constant by BNAH more inefficient (*k*_q_ ∼1.7 × 10^7^ M^–1^ s^–1^)^85^ than that of [Ru(bpy)_3_]^2+^ (*k*_q_ = 2.6 × 10^8^ M^–1^ s^–1^)^47^ by one order of magnitude or more.

**Fig. 7:**
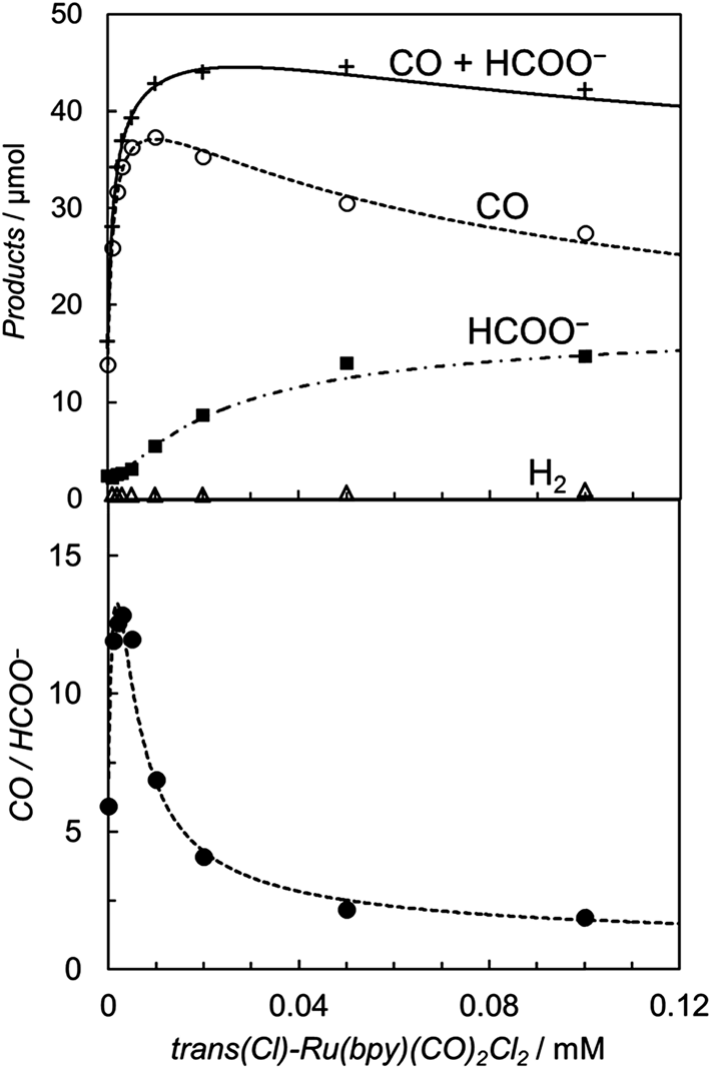
(Top) Plots of the amounts of the reduction products after 30 min of photo-irradiation (400 W Hg lamp, *λ* > 400 nm) *versus* the concentration of *trans*(Cl)–Ru(bpy)(CO)_2_Cl_2_ in CO_2_-saturated DMA/water (9 : 1, v/v) in the presence of [Ru(4dmbpy)_3_](PF_6_)_2_ (0.50 mM) and BNAH (0.10 M): CO (○), HCOO^–^ (■), H_2_ (Δ) and CO + HCOO^–^ (+). (Bottom) Plots of the CO/HCOO^–^ ratio *versus* the concentration of *trans*(Cl)–Ru(bpy)(CO)_2_Cl_2_. The curves represent the theoretical fittings based on the kinetic analyses (see eqn (3) and (4)).

Fig. 7 shows the relationship between the concentration of the catalyst and the initial rates for CO and formate production using [Ru(4dmbpy)_3_]^2+^. The simulation curves based on eqn (3) and (4) well reproduce the experimental plots, where formate shows an induction region for its production at very low concentration, and the plots for CO show a downward convex shape at high concentration. From the curve fittings in Fig. 7 and the blank experiment involving [Ru(4dmbpy)_3_]^2+^ the parameters are given as: *v*_0_ = 1.5 × 10^–6^ M s^–1^, *a*/*d* = 2.5 s^–1^, *b*/*d* = 1.0 × 10^10^ M^–2^, *c*/*d* = 7.7 × 10^5^ M^–1^, *v*_0_′ = 2.2 × 10^–7^ M s^–1^, *a*′/*d*′ = 1.5 × 10^4^ M^–1^ s^–1^, *b*′/*d*′ = 8.4 × 10^9^ M^–2^, *c*′/*d*′ = 2.1 × 10^5^ M^–1^. While the values of *b*/*c* and *b*′/*c*′, estimated to be 1.3 × 10^4^ M^–1^ and 4.0 × 10^4^ M^–1^, are similar to those using [Ru(bpy)_3_]^2+^ (*b*/*c* = 1.3 × 10^4^ M^–1^ and *b*′/*c*′ = 1.8 × 10^4^ M^–1^ in Fig. 3), the values of *a*/*c* and *a*′/*b*′, estimated to be 3.3 × 10^–6^ M s^–1^ and 1.8 × 10^–6^ M s^–1^, are smaller than those using [Ru(bpy)_3_]^2+^ (*a*/*c* = 1.2 × 10^–5^ M s^–1^ and *a*′/*b*′ = 0.6 × 10^–5^ M s^–1^ in Fig. 3). Assuming that the cage escape efficiency is the same as that for [Ru(bpy)_3_]^2+^ (*αI*_ex_ = 3.3 × 10^–5^ M s^–1^), the value of eqn (6) is estimated to be 9.4 × 10^–6^ M s^–1^ using the quenching rate constant of [Ru(4dmbpy)_3_]^2+^. Thus, the smaller values of *a*/*c* and *a*′/*b*′ estimated in Fig. 7 well reflect the smaller quenching rate constant (*k*_q_) of [Ru(4dmbpy)_3_]^2+^. In addition, the maximum selectivity attained for the products ratio is *ca.* 13 at the concentration of 3 μM of the catalyst, indicating that the selectivity of CO is higher than that observed in Fig. 3. The result implies that the reduction potential of Ru^+^ is more negative than that of Ru^2+^–CO in Scheme 4, and that the powerful reductant smoothly supplies the second electron to Ru^+^ in the low catalyst concentration region.

### Selective CO formation in photochemical CO_2_ reduction using *trans*(Cl)–Ru(6Mes-bpy)(CO)_2_Cl_2_

While we have succeeded in explaining the product selectivity with the kinetic analyses, we have not yet directly detected the dimer during the CO_2_ reduction reaction by means of spectroscopic methods such as UV-vis absorption and ESI-MS. This is because the absorption band of the dimer is overlapped with that of [Ru(bpy)_3_]^2+^ and the absorption coefficient of the dimer at 450 nm is around one-quarter smaller than that of [Ru(bpy)_3_]^2+^ (Fig. 4b).^86^ Even if all of the *trans*(Cl)–Ru(bpy)(CO)_2_Cl_2_ (0.1 mM) transforms into the Ru(i)–Ru(i) dimer during the photochemical reaction, the contribution of the dimer would be only 2.5% of the whole absorption. In addition, polymerization easily occurs even in CO_2_-saturated DMA/water when a high concentration (>0.2 mM) of *trans*(Cl)–Ru(bpy)(CO)_2_Cl_2_ is used. The attempt to detect the intermediate by ESI-MS has also failed so far due to the lower stability during the ionization process of the measurement. Thus, we changed the strategy to verify the proposed mechanism: we synthesized a novel ruthenium complex, *trans*(Cl)–Ru(6Mes-bpy)(CO)_2_Cl_2_, which has mesityl groups at the 6,6′-positions of the bipyridine ligand to suppress dimer formation, and investigated the product selectivity in the photocatalytic CO_2_ reduction.

**Fig. 8:**
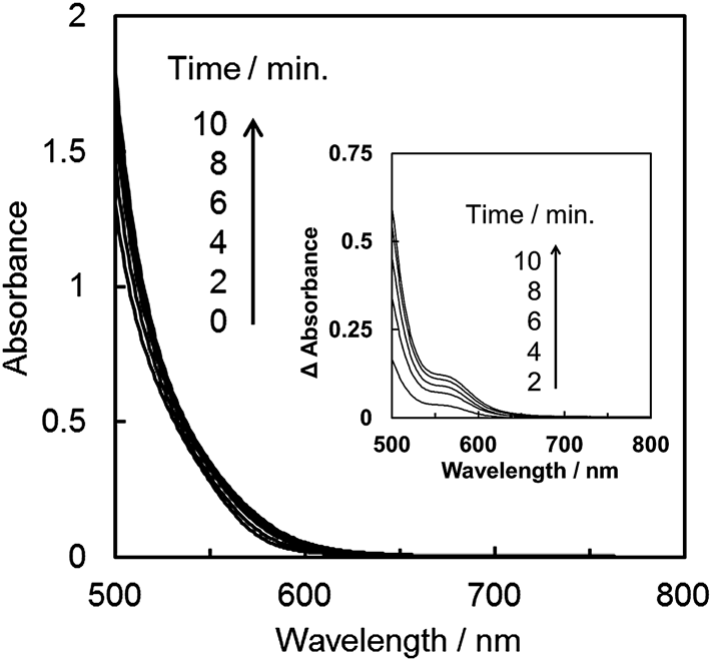
Absorption spectra of the DMA/water (9 : 1, v/v) solution containing *trans*(Cl)–Ru(6Mes-bpy)(CO)_2_Cl_2_ (0.20 mM), [Ru(bpy)_3_]^2+^ (0.50 mM) and BNAH (0.10 M) during the photo-irradiation with *λ* > 400 nm light with an intensity of 7.5 × 10^–7^ einstein s^–1^ under an Ar atmosphere. The inset shows the differential absorption spectra (optical path length: 10 mm).

The synthesis is described in the experimental section. The reduction potential of *trans*(Cl)–Ru(6Mes-bpy)(CO)_2_Cl_2_ (–1.56 V *vs.* Ag/Ag^+^ in DMA/water (9 : 1 v/v), Fig. S7 in ESI†) is similar to that of *trans*(Cl)–Ru(bpy)(CO)_2_Cl_2_, indicating that the mesityl groups do not strongly affect the electronic structure. This also suggests that the electron transfer reaction from the reduced photosensitizer to *trans*(Cl)–Ru(6Mes-bpy)(CO)_2_Cl_2_ occurs similarly to that of *trans*(Cl)–Ru(bpy)(CO)_2_Cl_2_. Before performing the photocatalytic CO_2_ reduction, we checked that *trans*(Cl)–Ru(6Mes-bpy)(CO)_2_Cl_2_ does not form a dimer by monitoring the absorption spectra of the solutions containing the ruthenium complex, [Ru(bpy)_3_]^2+^ and BNAH during photo-irradiation under an Ar atmosphere. In contrast to *trans*(Cl)–Ru(bpy)(CO)_2_Cl_2_, as shown in Fig. 4a, no absorption band corresponding to the polymeric complex is observed, as shown in Fig. 8, indicating that the bulky substituents at the 6,6′-positions of the bipyridyl ligand suppress the formation of the Ru–Ru bond. The differential absorption spectra show small changes to the spectra with a broad shoulder between 550 and 600 nm (see the inset in Fig. 8). They are comparable with the changes to the spectra in electrolysis (Fig. S8 in the ESI†), because in the spectra measured during the photo-reaction (Fig. 8), the absorptions of the photosensitizer and the electron donor overlap. The changes in the spectra during the photo-reaction would be due to formation of the one-electron reduced but non-dimerised species of the ruthenium complex.

**Fig. 9:**
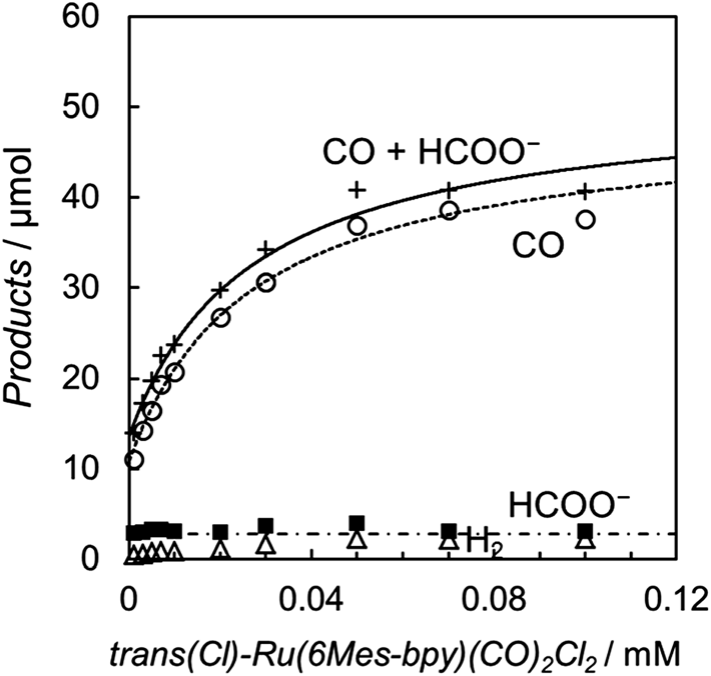
Plots of the amounts of the reduction products after 15 min of photo-irradiation (400 W Hg lamp, *λ* > 400 nm) *versus* the concentration of *trans*(Cl)–Ru(6Mes-bpy)(CO)_2_Cl_2_ in CO_2_-saturated DMA/water (9 : 1, v/v) in the presence of [Ru(bpy)_3_](PF_6_)_2_ (0.50 mM) and BNAH (0.10 M): CO (○), HCOO^–^ (■), H_2_ (Δ) and CO + HCOO^–^ (+). The curve for CO represents the theoretical fittings based on the kinetic analysis (see eqn (7)).

The photochemical CO_2_ reduction was carried out using *trans*(Cl)–Ru(6Mes-bpy)(CO)_2_Cl_2_ as the catalyst in a DMA/water (9 : 1 v/v) solution containing [Ru(bpy)_3_]^2+^ (0.50 mM) as the photosensitizer and BNAH (0.10 M) as the electron donor. The relationships between the concentration of the catalyst and the amounts of CO and formate are shown in Fig. 9. In contrast with Fig. 3, Fig. 9 shows that CO mainly forms, accompanied by a small amount of formate. The formate production is independent of the catalyst concentration, indicating that the formate comes from the blank reaction by [Ru(bpy)_3_]^2+^. The plots of the CO production *versus* the catalyst concentration are well fitted by the kinetic analysis based on the mechanism which does not include dimer formation. When the dimer formation is negligible in Scheme 3 and 4, the production rate for CO is expressed as eqn (7) (ESI†):7
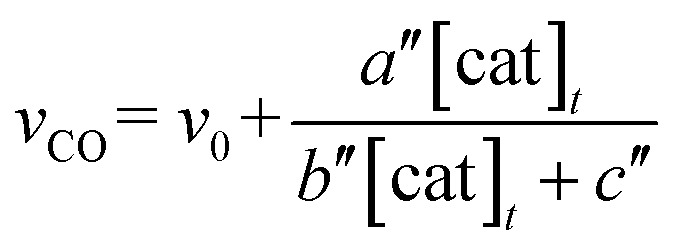
where *a*′′, *b*′′ and *c*′′ are the constant values as expressed by the following: *a*′′ = *k*_2_ *α k*_q_ [BNAH] *I*_ex_ *γ*, *b*′′ = 2 *k*_2_ (*k*_*n*+*nr*_ + *k*_q_ [BNAH])*γ*, *c*′′ = *k*_b_ (*k*_*n*+*nr*_ + *k*_q_ [BNAH]). Curve fitting based on eqn (6) gives the parameters: *v*_0_ = 2.4 × 10^–6^ M s^–1^, *a*′′/*b*′′ = 0.85 × 10^–5^ M s^–1^, *c*′′/*b*′′ = 2.7 × 10^–5^ M. The value of *a*′′/*b*′′ is also consistent with the value (1.6 × 10^–5^ M s^–1^) in eqn (6). Thus, *trans*(Cl)–Ru(6Mes-bpy)(CO)_2_Cl_2_, which does not form a dimer, affords CO selectively in the photocatalytic CO_2_ reduction.^87^

Deronzier and Ziessel *et al.* reported that electrochemical CO_2_ reduction using ruthenium polymers such as [Ru(bpy)(CO)_2_]_*n*_ affords CO selectively.^63–68^ These reports seem to be inconsistent with our results. It should be noted that the electrochemical reactions are different from the photochemical catalyses. In the electrochemical reaction, electrons can be efficiently supplied from the electrodes to the catalysts, resulting in the valences of the most ruthenium complexes being reduced to 0 or lower, to –1.^68^ On the contrary, in the photochemical catalyses discussed in this report, reduction of the catalysts occurs *via* the reaction between the reduced species of the photosensitizer and the catalyst. In the catalyses, there are various possible intermediates and reaction paths. The major reaction pathway is strongly dependent on the reaction (*e.g.*, photochemical or electrochemical reaction) and the conditions (*e.g.*, solvents, pH). The experimental results in this report suggest that the Ru(i) species of the dimer plays an important role in the reaction mechanism of formate production. It still remains unknown how the Ru(i) dimer can selectively produce formate from CO_2_. Further computational studies of the process are now under way.

## Conclusion

We have carried out photochemical CO_2_ reduction catalyzed by *trans*(Cl)–Ru(bpy)(CO)_2_Cl_2_, and have unexpectedly found that the product ratio of CO to formate depends on the concentration of the catalyst. In order to explain the behavior of the CO/HCOO^–^ selectivity, we have proposed a new reaction mechanism containing the formation of a catalyst dimer which selectively produces formate. The mechanism has strongly been supported by the kinetic analyses, the catalyses by [Ru(bpy)(CO)_2_Cl]_2_ and the light intensity dependence of the CO/HCOO^–^ selectivity. The mechanism of the photocatalytic CO_2_ reduction consists of the electron relay cycle and the catalytic CO_2_ reduction cycle. The former is the process in which the reduced photosensitizer (PS^–^) supplies electrons to the catalyst, and the latter is the steps where the ruthenium complexes catalytically reduce CO_2_ by using the supplied electrons. At high catalyst concentration, the electron relay system would be rate-determining because the catalysis becomes faster than the electron supply. Under this condition, the ruthenium catalyst cannot be supplied with sufficient electrons; the one-electron reduced species of the catalyst is not able to receive more electrons, and it forms the dimer, which produces HCOO^–^. Therefore as the catalyst concentration increases, the product selectivity (CO/HCOO^–^) decreases. As the light intensity is reduced, the concentration of PS^–^ also decreases, resulting in the same effect as a high concentration of the catalyst. The mechanism also explains the photo-irradiation time dependence of the CO_2_ reduction: the CO production reaches saturation. At longer reaction times, the electron donor BNAH is consumed to decrease the concentration of PS^–^. In this situation, the one-electron reduced species of the catalyst is not able to receive more electrons and forms the catalyst dimer, making the CO production decrease. However, the HCOO^–^ formation continues until the electron donor is exhausted. We have further designed and synthesized a novel ruthenium complex, *trans*(Cl)–Ru(6Mes-bpy)(CO)_2_Cl_2_, which has a bulky ligand to eliminate the contribution of the dimer. By suppressing the dimer formation, the photochemical CO_2_ reduction produces CO selectively. Among photocatalytic systems for the CO_2_ reduction by ruthenium complexes, to the best of our knowledge, this system is the first case producing CO selectively. This finding not only elucidates the reaction mechanisms for the photocatalytic CO_2_ reduction but also leads us to design more effective metal complexes for the catalyses.
