# Supplementary material for: Unexpected effect of catalyst concentration on photochemical CO2 reduction by trans(Cl)–Ru(bpy)(CO)2Cl2: new mechanistic insight into the CO/HCOO– selectivity
Source: Chem Sci. 2015 Mar 12;6(5):3063–74. doi: 10.1039/c5sc00199d (PMC5490049; doi:10.1039/c5sc00199d)
Supplement: Supplementary file 1 [file SC-006-C5SC00199D-s001.pdf]

Electronic Supplementary Material (ESI) for Chemical Science.
This journal is © The Royal Society of Chemistry 2015

Supporting Information

Unexpected effect of catalyst concentration on photochemical CO_2 reduction by *trans*(Cl)-Ru(bpy)$(CO)_2Cl_2$: new mechanistic insight into the CO/$HCOO^-$ selectivity

Yusuke Kuramochi,[†] Jun Itabashi,[†] Kyohei Fukaya,[†] Akito Enomoto,[†] Makoto Yoshida,[†] and Hitoshi Ishida[†, ‡]*

[†] Department of Chemistry, Graduate School of Science, Kitasato University, 1-15-1 Kitasato, Minami-ku, Sagamihara, Kanagawa 252-0373, Japan

[‡] Precursory Research for Embryonic Science (PRESTO), Japan Science and Technology Agency (JST), 4-1-8 Honcho, Kawaguchi, Saitama 332-0012, Japan

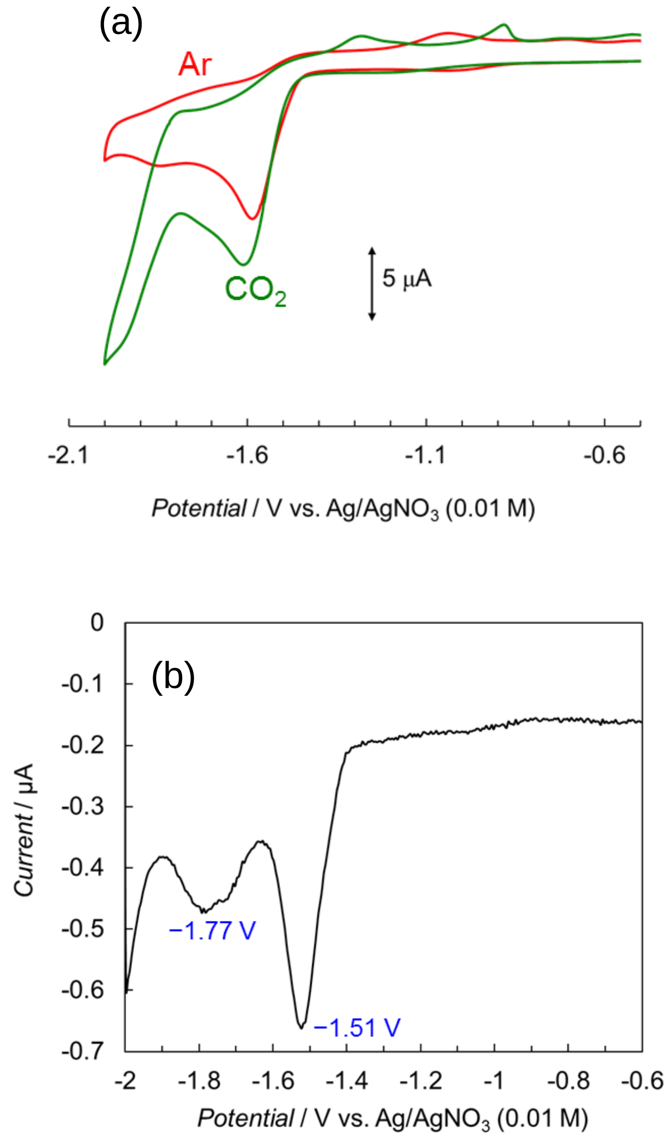

Figure. S1. (a) Cyclic voltammograms (CVs) of *trans*(Cl)-Ru(bpy)$(CO)_2Cl_2$ (0.50 mM) under Ar (red) and CO_2 (green) in DMA/water (9:1 v/v) using 0.10 M ^{n}Bu_4NClO_4 as the supporting electrolyte and Ag/$AgNO_3$ (1.0×10^{-2} M, in CH_3CN) as the reference electrode. Scan rate: 100 mV/s. (b) Differential pulse voltammogram (DPV) of *trans*(Cl)-Ru(bpy)$(CO)_2Cl_2$ (0.50 mM) in the Ar-saturated DMA/water (9:1 v/v) solution.

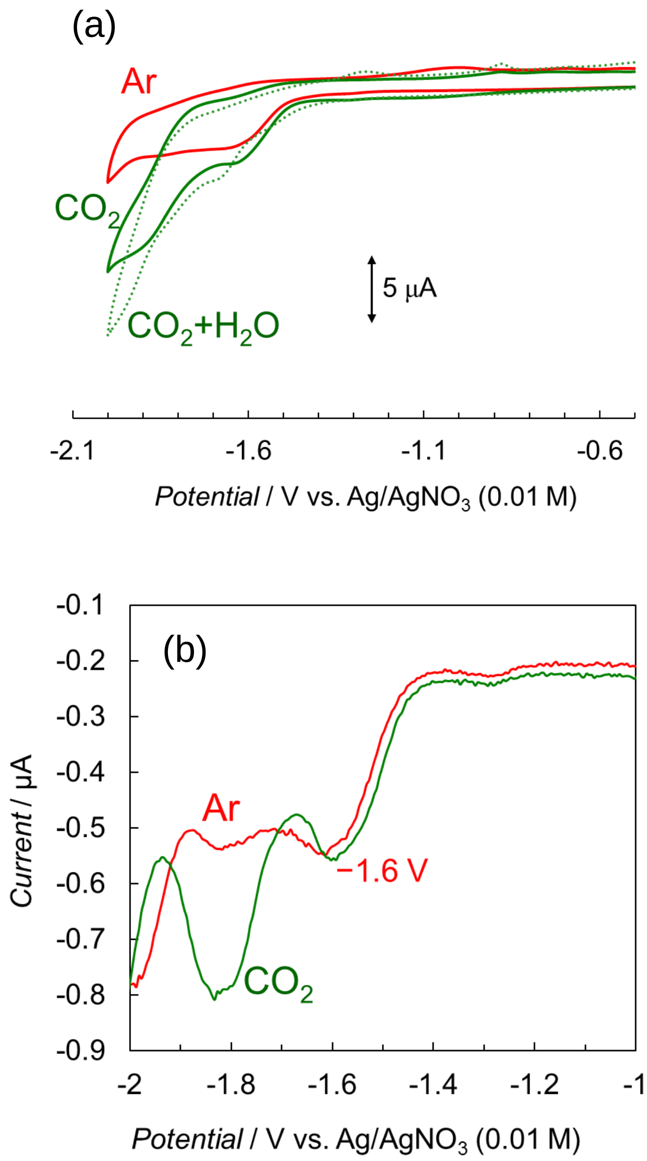

Figure. S2. (a) Cyclic voltammograms (CVs) of $[Ru(bpy)(CO)_2Cl]_2$ (0.30 mM) under Ar (red), CO_2 (green solid) and CO_2 + 10w% H_2O (green dotted) in DMA using 0.10 M $^{n}Bu_4NClO_4$ as the supporting electrolyte and Ag/$AgNO_3$ (1.0×10^{-2} M, in CH_3CN) as the reference electrode. Scan rate: 100 mV/s. (b) Differential pulse voltammograms (DPV) of $[Ru(bpy)(CO)_2Cl]_2$ (0.30 mM) under Ar (red) and CO_2 (green) in DMA.

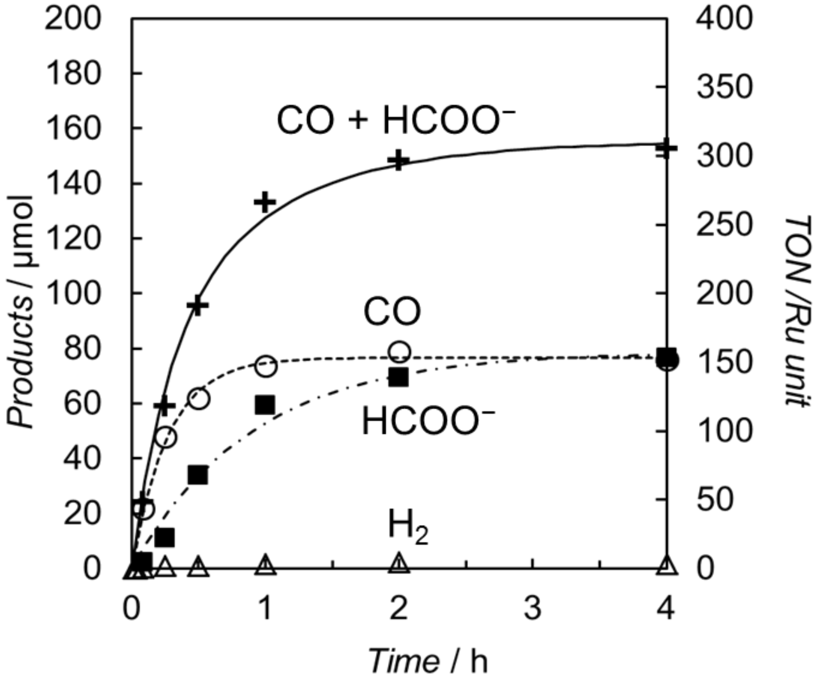

Figure S3. Photo-irradiation time dependence of the products in the CO_2-saturated DMA/water (9:1 v/v) solution containing $[Ru(bpy)(CO)_2Cl]_2$ (0.050 mM), $[Ru(bpy)_3](PF_6)_2$ (0.50 mM) and BNAH (0.10 M): CO (○), $HCOO^-$ (■), H_2 (Δ) and CO + $HCOO^-$ (+).

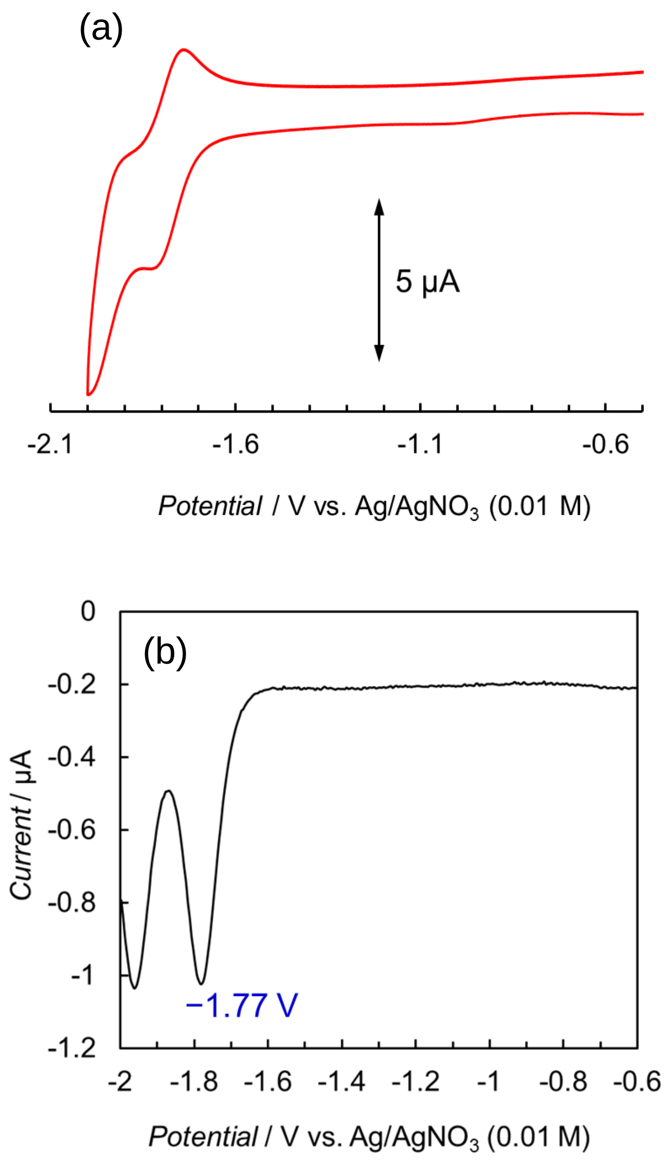

Figure. S4. (a) Cyclic voltammogram (CV) of [Ru(4dmbpy)$_3$](PF$_6$)$_2$ (0.50 mM) under Ar in DMA/water (9:1 v/v) using 0.10 M ^{n}Bu$_4$NClO$_4$ as the supporting electrolyte and Ag/AgNO$_3$ (1.0×10^{-2} M, in CH$_3$CN) as the reference electrode. Scan rate: 100 mV/s. (b) Differential pulse voltammogram (DPV) of [Ru(4dmbpy)$_3$](PF$_6$)$_2$ (0.50 mM) in the Ar-saturated DMA/water (9:1 v/v) solution.

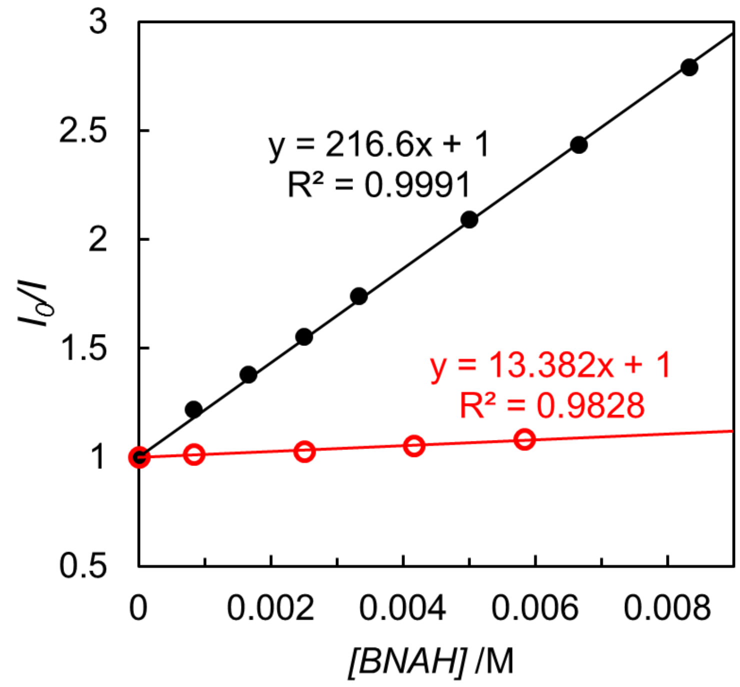

Figure. S5. Stern-Volmer plots for emission quenchings of the excited (black) $[Ru(bpy)_3]^{2+}$ and (red) $[Ru(4dmbpy)_3]^{2+}$ by BNAH in DMA/water (9:1 v/v). See the reference: Y. Kuramochi, M. Kamiya, H. Ishida, *Inorg. Chem.* 2014, **53**, 3326-3332.

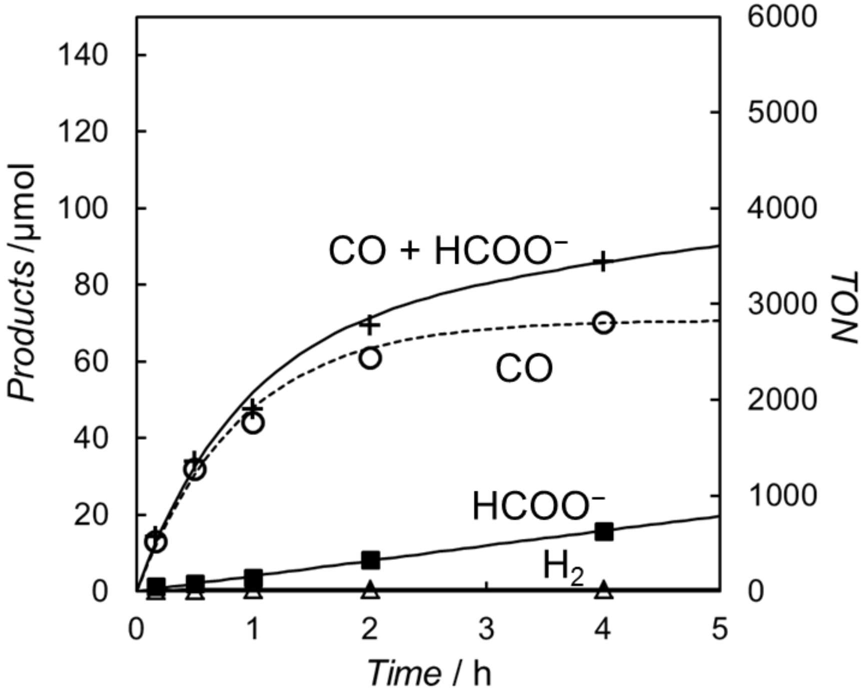

Figure S6. Photo-irradiation time dependence of the products in the CO_2-saturated DMA/water (9:1 v/v) solution containing *trans*(Cl)-Ru(bpy)$(CO)_2Cl_2$ (5.0 μM), [Ru(4dmbpy)$_3$]$(PF_6)_2$ (0.50 mM) and BNAH (0.10 M): CO (○), $HCOO^-$ (■), H_2 (Δ) and CO + $HCOO^-$ (+).

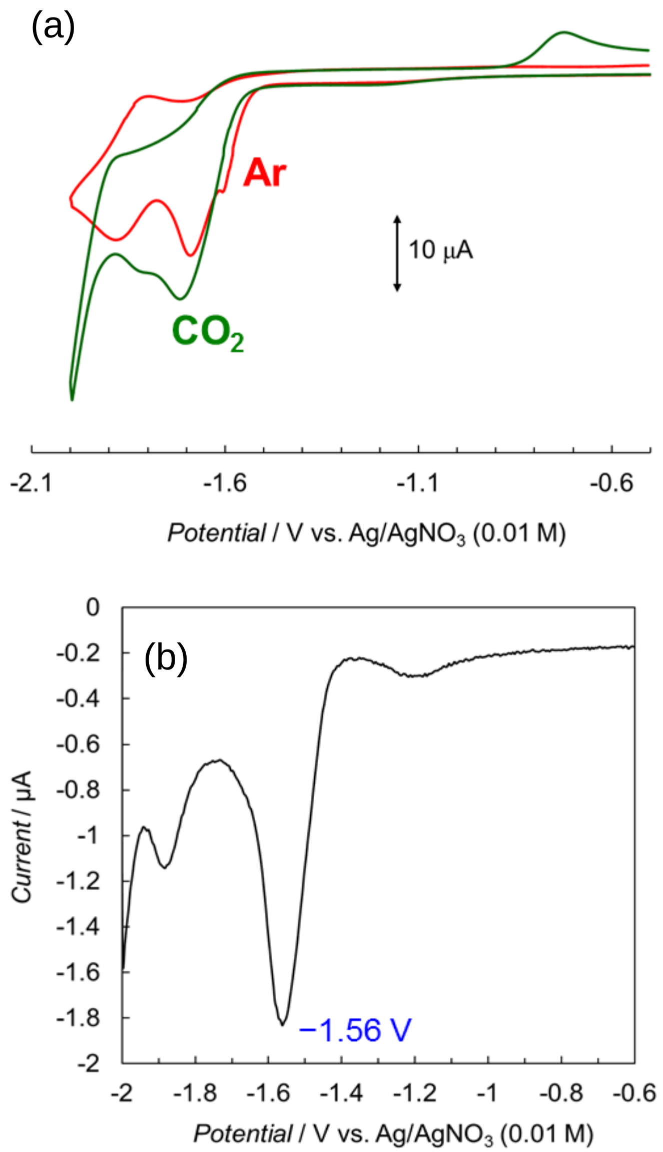

Figure. S7. (a) Cyclic voltammograms (CVs) of *trans*(Cl)-Ru(6Mes-bpy)$(CO)_2Cl_2$ (1.0 mM) under Ar (red) and CO_2 (green) in DMA/water (9:1 v/v) using 0.10 M ^{n}Bu$_4$NClO$_4$ as the supporting electrolyte and Ag/AgNO$_3$ (1.0×10^{-2} M, in CH$_3$CN) as the reference electrode. Scan rate: 100 mV/s. (b) Differential pulse voltammogram (DPV) of *trans*(Cl)-Ru(6Mes-bpy)$(CO)_2$-Cl_2 (1.0 mM) in the Ar-saturated DMA/water (9:1 v/v) solution.

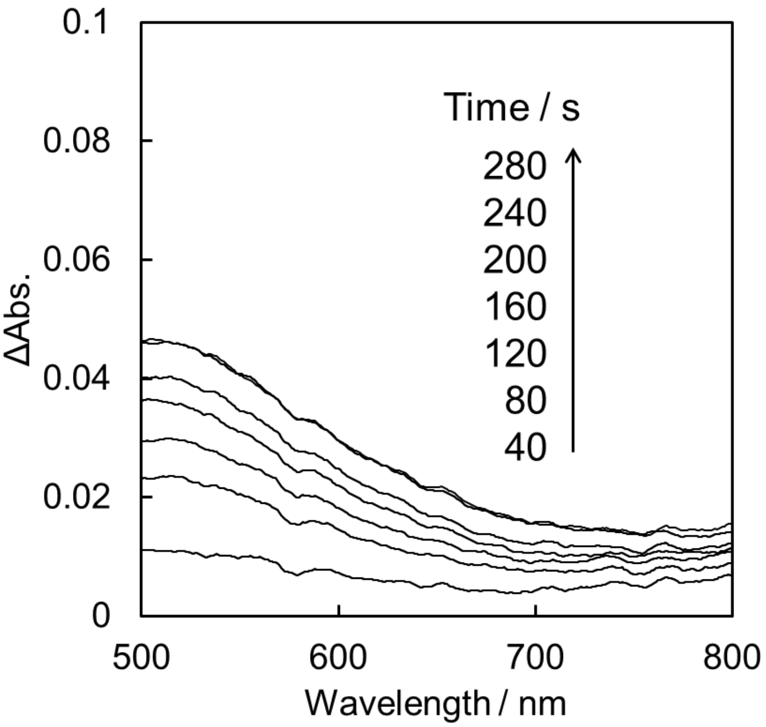

Figure S8. Absorption spectral changes of the Ar-saturated DMA solution of *trans*(Cl)-Ru(6Mes-bpy)$(CO)_2Cl_2$ (1.0 mM) and $^{n}Bu_4NClO_4$ (0.10 M) under the controlled potential electrolysis at −1.70 V vs. Ag/Ag^+ (optical path length: 1 mm); the absorption raised at longer wavelengths might be due to bubbles formed during the electrolysis.

Kinetic Analyses

From Scheme 3 in the main text, the steady state approximation affords the PS and PS* concentrations as expressed as equations (S1) and (S2), respectively.

$$\frac{d[\mathrm{PS}]}{dt} = -I_{ex} + k_{r+nr}[\mathrm{PS}^*] + \beta\, k_q\, [\mathrm{BNAH}][\mathrm{PS}^*] + k_b[\mathrm{PS}^-] + (\textstyle\sum_i k_i\, [\mathrm{cat}_i])[\mathrm{PS}^-] = 0 \qquad \text{(S1)}$$

$$\frac{d[\mathrm{PS}^*]}{dt} = I_{ex} - k_{r+nr}[\mathrm{PS}^*] - (\alpha + \beta) k_q\, [\mathrm{BNAH}][\mathrm{PS}^*] = 0 \qquad \text{(S2)}$$

Combining equations (S1) and (S2):

$$k_b[\mathrm{PS}^-] + (\textstyle\sum_i k_i\, [\mathrm{cat}_i])[PS^-] - \alpha\, k_q\, [\mathrm{BNAH}][\mathrm{PS}^*] = 0$$

$$[\mathrm{PS}^-] = \frac{\alpha\, k_q\, [\mathrm{BNAH}]}{\{k_b + (\sum_i k_i [\mathrm{cat}_i])\}} [\mathrm{PS}^*] \qquad \text{(S3)}$$

From equation (S2):

$$[\mathrm{PS}^*] = \frac{I_{ex}}{k_{r+nr} + k_q\, [\mathrm{BNAH}]} \qquad \text{(S4)}$$

Combining equations (S3) and (S4):

$$[\mathrm{PS}^-] = \frac{\alpha\, k_q\, [\mathrm{BNAH}]\ I_{ex}}{k_b(k_{r+nr} + k_q\, [\mathrm{BNAH}]) + \sum_i k_i [\mathrm{cat}_i](k_{r+nr} + k_q\, [\mathrm{BNAH}])} \qquad \text{(S5)}$$

$$= \frac{(\alpha\, k_q\, [\mathrm{BNAH}]\ I_{ex})/\{k_b(k_{r+nr} + k_q\, [\mathrm{BNAH}])\}}{1 + \sum_i k_i [\mathrm{cat}_i]/k_b} \qquad \text{(2)}$$

According to Scheme 4 in the main text, contribution of the electron transfer from $[Ru(bpy)_3]^+$ to the catalyst in equation (2) is expressed as equation (S6) using the steady state approximation:

$$\sum_i k_i[\mathrm{cat}_i] = k_1[\textbf{Ru-CO}^{2+}] + k_2[\textbf{Ru}^+] + k_3[\textbf{Ru}^+ - \textbf{Ru}^+] + k_4[\textbf{Ru}^+ - \textbf{Ru-OCOH}^+]$$

$$= k_1\frac{k_2}{k_1}[\textbf{Ru}^+] + k_2[\textbf{Ru}^+] + k_3\frac{k_d[\textbf{Ru}^+]^2}{k_{-d}+k_3[\mathrm{PS}^-]} + k_4\frac{k_3k_d[\textbf{Ru}^+]^2}{k_4(k_{-d}+k_3[\mathrm{PS}^-])}$$

$$= 2\,k_2[\textbf{Ru}^+] + \frac{2\,k_3k_d[\textbf{Ru}^+]^2}{k_{-d}+k_3[\mathrm{PS}^-]} \qquad \text{(S6)}$$

Combining equations (2) and (S6):

$$[\mathrm{PS}^-] = \frac{(\alpha\, k_q\,[\mathrm{BNAH}]\; I_{ex})/(k_{r+nr}+k_q\,[\mathrm{BNAH}])\,(k_{-d}+k_3[\mathrm{PS}^-])}{(k_b+2k_2[\textbf{Ru}^+])(k_{-d}+k_3[\mathrm{PS}^-])+2k_d[\textbf{Ru}^+]^2k_3} \qquad \text{(S7)}$$

Since $[PS^-] << 1$ M, equation (S7) would become:

$$[\mathrm{PS}^-] = \frac{\dfrac{k_{-d}\,\alpha\, k_q\,[\mathrm{BNAH}]\; I_{ex}}{k_{r+nr}+k_q\,[\mathrm{BNAH}]}}{2k_dk_3[\textbf{Ru}^+]^2 + 2k_{-d}k_2[\textbf{Ru}^+] + k_bk_{-d} + \dfrac{k_3\,\alpha\, k_q\,[\mathrm{BNAH}]\; I_{ex}}{k_{r+nr}+k_q\,[\mathrm{BNAH}]}} \qquad \text{(S8)}$$

The initial rate for CO production is:

$$\frac{d[\mathrm{CO}]}{dt} = k_1[\textbf{Ru-CO}^{2+}][\mathrm{PS}^-] = k_2[\textbf{Ru}^+][\mathrm{PS}^-] \qquad \text{(S9)}$$

Combining equations (S8) and (S9):

$$\frac{d[\mathrm{CO}]}{dt} = \frac{k_2\,\dfrac{k_{-d}\,\alpha\, k_q\,[\mathrm{BNAH}]\; I_{ex}}{k_{r+nr}+k_q\,[\mathrm{BNAH}]}\,[\textbf{Ru}^+]}{2k_dk_3[\textbf{Ru}^+]^2 + 2k_{-d}k_2[\textbf{Ru}^+] + k_bk_{-d} + \dfrac{k_3\,\alpha\, k_q\,[\mathrm{BNAH}]\; I_{ex}}{k_{r+nr}+k_q\,[\mathrm{BNAH}]}} \qquad \text{(S10)}$$

When [$\mathbf{Ru}^+$] << 1 M, [$\mathbf{Ru}^+$] = γ [cat]$_t$, where [cat]$_t$ is the initial concentration of *trans*(Cl)-Ru(bpy)(CO)$_2$Cl$_2$, γ is a proportional constant peculiar to the catalyst. The value of γ would be related to k_{CO2} and k_{CO2}', and a larger γ is expected to indicate higher reaction rate of the reduced catalyst with CO_2 and H^+. Equation (S10) can be written:

$$\frac{d[\mathrm{CO}]}{dt} = \frac{a[\mathrm{cat}]_t}{b[\mathrm{cat}]_t^{\;2} + c[\mathrm{cat}]_t + d} = \frac{(a/d)[\mathrm{cat}]_t}{(b/d)[\mathrm{cat}]_t^{\;2} + (c/d)[\mathrm{cat}]_t + 1} \qquad \text{(S11)}$$

where *a*, *b*, *c* and *d* are expressed as the following:

$$a = k_2 \frac{k_{-d}\, \alpha\, k_q\, [\mathrm{BNAH}]\; I_{ex}}{k_{r+nr} + k_q\, [\mathrm{BNAH}]} \gamma \qquad \text{(S12)}$$

$$b = 2k_d k_3\, \gamma^{\,2} \qquad \text{(S13)}$$

$$c = 2k_{-d} k_2\, \gamma \qquad \text{(S14)}$$

$$d = k_b k_{-d} + \frac{k_3\, \alpha\, k_q\, [\mathrm{BNAH}]\; I_{ex}}{k_{r+nr} + k_q\, [\mathrm{BNAH}]} \qquad \text{(S15)}$$

Considering the blank products caused by [Ru(bpy)$_3$](PF$_6$)$_2$, equation (S11) becomes equation (3) in the main text. v_0 is the blank formation rate of CO.

$$v_{CO}(\mathrm{M/s}) = v_0(\mathrm{M/s}) + \frac{a\,[\mathrm{cat}]_t\,(\mathrm{M})}{b\,[\mathrm{cat}]_t^{\;2}(\mathrm{M}^2) + c\,[\mathrm{cat}]_t(\mathrm{M}) + d} \qquad \text{(3)}$$

Curve fitting on the experimental result in Figure 3 gives a following equation with specific values of *a*, *b*, *c* and *d* (see Figure S9).

$$v_{CO}(\mathrm{M/s}) = 1.2 \times 10^{-6} + \frac{1.1[\mathrm{cat}]_t\,(\mathrm{M})}{1.1{\times}10^{9}[\mathrm{cat}]_t^{\;2}(\mathrm{M}^2) + 9.1{\times}10^{4}[\mathrm{cat}]_t(\mathrm{M}) + 1} \qquad \text{(S16)}$$

where concentration of CO is calculated by dividing mol of CO with volume of the reaction solution (5.0 mL).

The initial rate for formate production is:

$$\frac{d[\mathrm{HCOO^-}]}{dt} = k_4[\mathbf{Ru^+ - Ru\text{-}OCOH^+}][\mathrm{PS^-}] = k_3 \frac{k_d[\mathbf{Ru^+}]^2}{k_{-d}+k_3[\mathrm{PS^-}]}[\mathrm{PS^-}]$$

$$= k_3 \frac{k_d[\mathbf{Ru^+}]^2}{k_{-d}/[\mathrm{PS^-}]+k_3} \qquad \text{(S17)}$$

Combining equations (S8) and (S17):

$$\frac{d[\mathrm{HCOO^-}]}{dt} = \frac{k_3k_d \frac{\alpha\, k_q\,[\mathrm{BNAH}]\; I_{ex}}{k_{r+nr}+k_q\,[\mathrm{BNAH}]}[\mathbf{Ru^+}]^2}{2k_dk_3\,[\mathbf{Ru^+}]^2 + 2k_{-d}k_2[\mathbf{Ru^+}] + k_bk_{-d}} \qquad \text{(S18)}$$

When $[\mathrm{Ru^+}] = \gamma\,[\mathrm{cat}]_t$, equation (S18) can be written:

$$\frac{d[\mathrm{HCOO^-}]}{dt} = \frac{a'[\mathrm{cat}]_t^{\,2}}{b'[\mathrm{cat}]_t^{\,2} + c'[\mathrm{cat}]_t + d'} \qquad \text{(S19)}$$

where *a'*, *b'*, *c'* and *d'* are expressed as the following:

$$a' = k_3k_d \frac{\alpha\, k_q\,[\mathrm{BNAH}]\; I_{ex}}{k_{r+nr}+k_q\,[\mathrm{BNAH}]}\gamma^{\,2} \qquad \text{(S20)}$$

$$b' = 2k_dk_3\,\gamma^{\,2} \qquad \text{(S21)}$$

$$c' = 2k_{-d}k_2\,\gamma \qquad \text{(S22)}$$

$$d' = k_bk_{-d} \qquad \text{(S23)}$$

Considering the blank products caused by $[\mathrm{Ru(bpy)_3}](\mathrm{PF_6})_2$, equation (S19) becomes equation (4) in the main text. v_0' is the blank formation rate of formate.

$$v_{HCOO^-}(\mathrm{M/s}) = v_0'\,(\mathrm{M/s}) + \frac{a'[\mathrm{cat}]_t^{\,2}\,(\mathrm{M})}{b'[\mathrm{cat}]_t^{\,2}(\mathrm{M^2}) + c'[\mathrm{cat}]_t(\mathrm{M}) + d'} \qquad (4)$$

Concentration of formate is calculated by dividing mol of formate with volume of the reaction solution (5.0 mL). The fitting analyses show that *a'*, *b'* and *c'* have specific values while the simulation curve is less dependent on the value of *d'* (see Figure S10). When the term of *d'* is negligible, equation (4) becomes:

$$v_{HCOO^-}(\mathrm{M/s}) = v_0' \,(\mathrm{M/s}) + \frac{a' \,[\mathrm{cat}]_t(\mathrm{M})}{b' \,[\mathrm{cat}]_t(\mathrm{M}) + c'} \quad (5)$$

Double-reciprocal form becomes:

$$(v_{HCOO^-} - v_0')^{-1} = \frac{c'}{a'}\frac{1}{[\mathrm{cat}]_t} + \frac{b'}{a'} \quad (\mathrm{S24})$$

Double-reciprocal plots give a following equation.

$$v_{HCOO^-}(\mathrm{M/s}) = 4.2 \times 10^{-7} + \frac{0.10[\mathrm{cat}]_t\,(\mathrm{M})}{1.8\times10^{4}[\mathrm{cat}]_t\,(\mathrm{M}) + 1} \quad (\mathrm{S25})$$

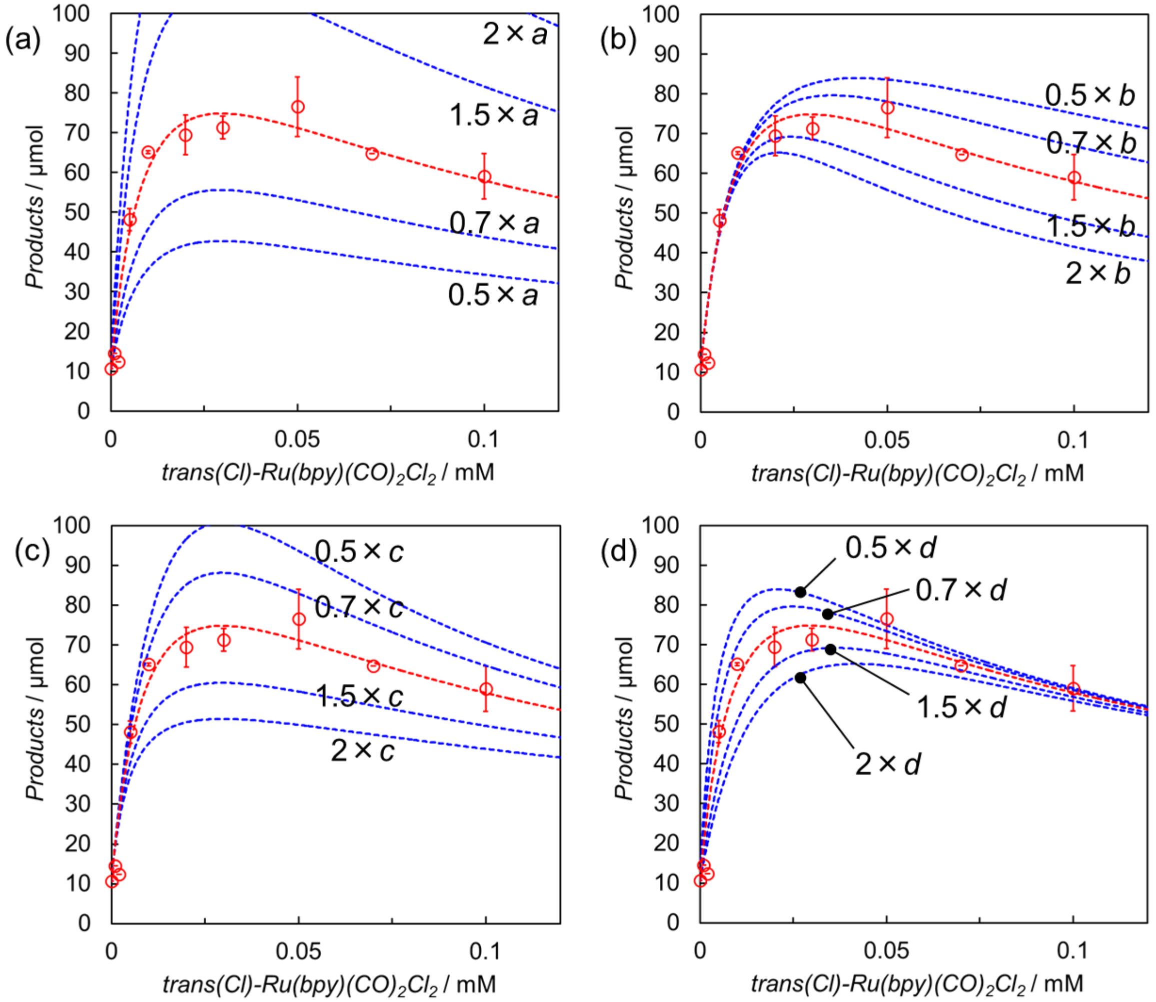

Figure S9. Simulation curves by changing the parameters of (a) *a*, (b) *b*, (c) *c* and (d) *d* in equation (3). The red lines show the simulation curves using $a = 1.1$, $b = 1.1\times10^9$, $c = 9.1\times10^4$ and $d = 1.0$.

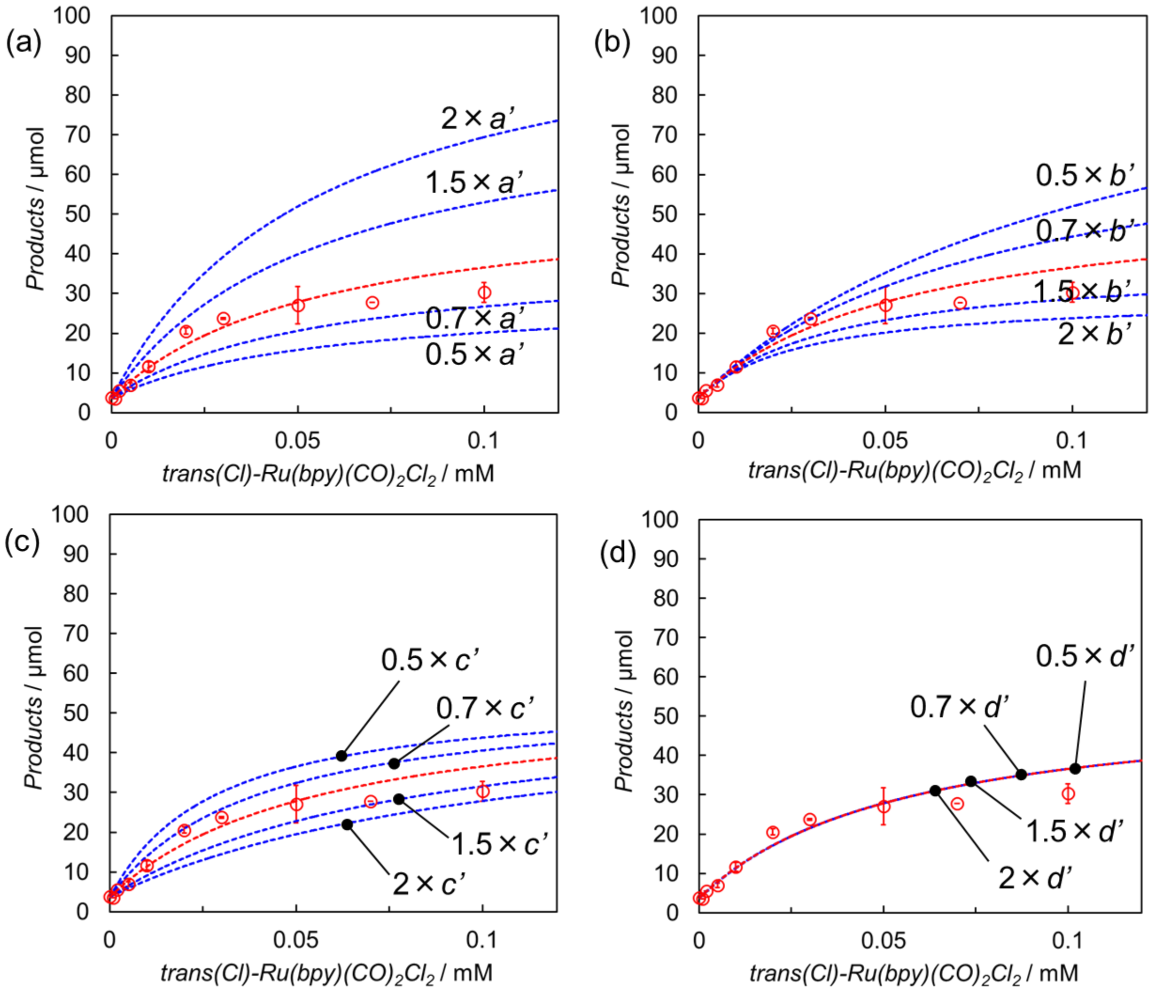

Figure S10. Simulation curves by changing the parameters of (a) *a'*, (b) *b'*, (c) *c'* and (d) *d'* in equation (4). The red lines show the simulation curves using $a' = 1.0\times10^6$, $b' = 1.8\times10^{11}$, $c' = 1.0\times10^7$ and $d' = 1.0$.

Kinetic Analyses without Forming the Dimer

When the dimer formation is negligible in Scheme 4, equation (S6) becomes:

$$\sum_i k_i[\mathrm{cat}_i] = k_1[\mathbf{Ru\text{-}CO^{2+}}] + k_2[\mathbf{Ru^+}] \quad = 2\, k_2[\mathbf{Ru^+}] \qquad \text{(S26)}$$

Combining equations (2) and (S26):

$$[\mathrm{PS}^-] = \frac{\alpha\, k_q\, [\mathrm{BNAH}]\ I_{ex}}{2k_2(k_{r+nr}+k_q\, [\mathrm{BNAH}])[\mathbf{Ru^+}] + k_b(k_{r+nr}+k_q\, [\mathrm{BNAH}])} \qquad \text{(S27)}$$

Combining equations (S9) and (S27) and considering the blank product:

$$v_{CO}(\mathrm{M/s}) = v_0(\mathrm{M/s}) + \frac{a''[\mathrm{cat}]_t}{b''[\mathrm{cat}]_t + c''} \qquad \text{(7)}$$

where *a''*, *b''* and *c''* are expressed as the following:

$$a'' = k_2\, \alpha\, k_q\, [\mathrm{BNAH}]\ I_{ex}\ \gamma \qquad \text{(S28)}$$

$$b'' = 2k_2(k_{r+nr} + k_q\, [BNAH])\, \gamma \qquad \text{(S29)}$$

$$c'' = k_b(k_{r+nr} + k_q\, [BNAH]) \qquad \text{(S30)}$$
